# Cofactor composition and function of a H_2_-sensing regulatory hydrogenase as revealed by Mössbauer and EPR spectroscopy†
†Electronic supplementary information (ESI) available: Tables with the simulation parameters and details of the Mössbauer, and EPR spectra (Tables S1–S4). additional EPR and Mössbauer spectra in Fig. S1–S9. See DOI: 10.1039/c5sc01560j



**DOI:** 10.1039/c5sc01560j

**Published:** 2015-05-26

**Authors:** Federico Roncaroli, Eckhard Bill, Bärbel Friedrich, Oliver Lenz, Wolfgang Lubitz, Maria-Eirini Pandelia

**Affiliations:** a Max-Planck-Institut für Chemische Energiekonversion , Stiftstraße 34-36 , 45470 Mülheim an der Ruhr , Germany . Email: wolfgang.lubitz@cec.mpg.de ; Email: eckhard.bill@cec.mpg.de; b Department of Condensed Matter Physics , Centro Atómico Constituyentes , Comisión Nacional de Energía Atómica (CNEA) , Argentina; c Institut für Biologie/Mikrobiologie , Humboldt-Universität zu Berlin , Chausseestraße 117 , 10115 Berlin , Germany; d Institut für Chemie , Technische Universität Berlin , Max-Volmer-Laboratorium , Straße des 17. Juni 135 , 10623 Berlin , Germany; e The Pennsylvania State University , Department of Chemistry , State College , PA 16802 , USA . Email: mxp65@psu.edu

## Abstract

The regulatory hydrogenase (RH) from *Ralstonia eutropha* H16 acts as a sensor for the detection of environmental H_2_ and regulates gene expression related to hydrogenase-mediated cellular metabolism. In marked contrast to prototypical energy-converting [NiFe] hydrogenases, the RH is apparently insensitive to inhibition by O_2_ and CO. While the physiological function of regulatory hydrogenases is well established, little is known about the redox cycling of the [NiFe] center and the nature of the iron–sulfur (FeS) clusters acting as electron relay. The absence of any FeS cluster signals in EPR had been attributed to their particular nature, whereas the observation of essentially only two active site redox states, namely Ni-SI and Ni-C, invoked a different operant mechanism. In the present work, we employ a combination of Mössbauer, FTIR and EPR spectroscopic techniques to study the RH, and the results are consistent with the presence of three [4Fe–4S] centers in the small subunit. In the as-isolated, oxidized RH all FeS clusters reside in the EPR-silent 2+ state. Incubation with H_2_ leads to reduction of two of the [4Fe–4S] clusters, whereas only strongly reducing agents lead to reduction of the third cluster, which is ascribed to be the [4Fe–4S] center in ‘proximal’ position to the [NiFe] center. In the two different active site redox states, the low-spin Fe^II^ exhibits distinct Mössbauer features attributed to changes in the electronic and geometric structure of the catalytic center. The results are discussed with regard to the spectral characteristics and physiological function of H_2_-sensing regulatory hydrogenases.

## Abstract 2

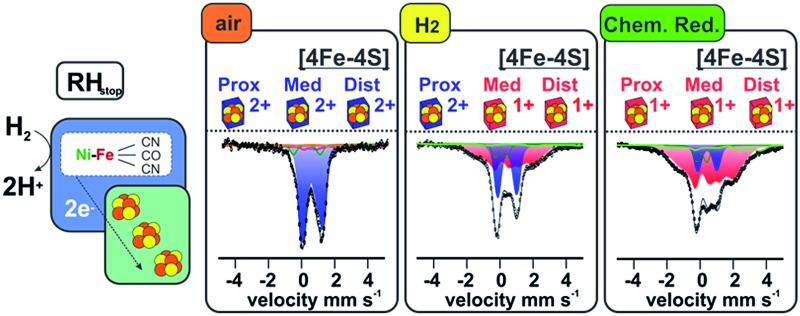
A regulatory hydrogenase is characterised by Mössbauer, EPR and FTIR yielding insight into structure and function of this dihydrogen sensor.

## Introduction

Hydrogenases, the biocatalysts carrying out the reversible oxidation of H_2_ in nature, are complex metalloenzymes, which harbor metal cofactors with a fascinating coordination chemistry.^1–4^ According to their active site metal content, they are grouped into [NiFe]-, [FeFe]- and [Fe]-hydrogenases.^4^ The enzyme investigated in this study belongs to the class of [NiFe]-hydrogenases, whose basic module consists of two subunits. The large subunit harbors the heterobimetallic [NiFe] center, whereas the small subunit carries up to three iron–sulfur clusters, which serve as an electron relay during catalysis.^4–8^

[NiFe]-hydrogenases are classified into five phylogenetically distinct groups on the basis of their structure and cellular function.^9,10^ Although most [NiFe]-hydrogenases are strongly inhibited by molecular oxygen, at least four of the subfamilies contain members that retain catalytic activity in the presence of O_2_,^10,11^ which is in particular a most interesting property with respect to any potential application for photocatalytic hydrogen production.^4^ Both the degree and the biophysical/biochemical origin of this O_2_-tolerance differ between the different subgroups. In selected representatives of group 1, O_2_-tolerance is crucially linked to the presence of a novel [4Fe–3S] cluster with unprecedented redox properties.^7,8,12–16^ H_2_ sensors belong to the group 2 of hydrogenases and are virtually resistant towards O_2_ and CO. This property has been primarily associated with a narrow intramolecular hydrophobic gas channel that impedes O_2_ diffusion to the [NiFe] site.^17–19^

**Fig. 1:**
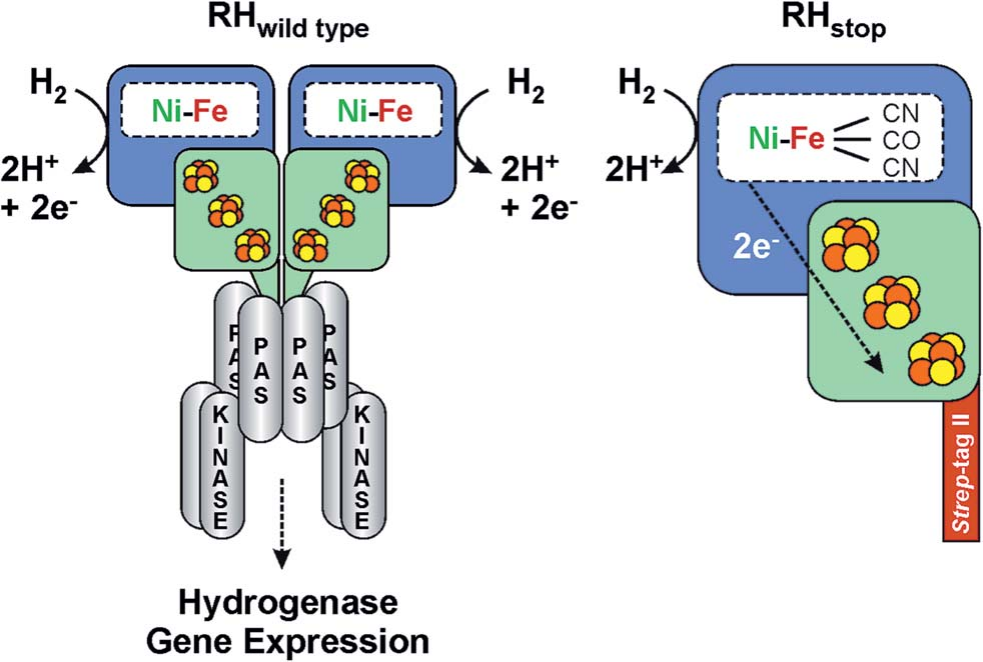
Scheme of the heterodimeric wild type RH hydrogenase in complex with the histidine kinase (left) and the RH_stop_ derivative employed in this study (right). See also ref. 36.

The facultatively lithoautotrophic β-proteobacterium *Ralstonia eutropha* (*Re*) H16 harbors four different O_2_-tolerant [NiFe] hydrogenases: a membrane-bound hydrogenase (MBH), a NAD^+^-reducing soluble hydrogenase (SH), an actinobacterial-type hydrogenase (AH) and a regulatory hydrogenase (RH).^10,11,19–26^ The RH is a group 2b protein and acts as H_2_ sensor in a signal transduction pathway, which ensures that the energy-generating hydrogenases, MBH and SH, are only synthesized if their substrate H_2_, is available.^9,24^ The H_2_-sensing unit is composed of two RH heterodimers that are tightly connected to a histidine protein kinase homotetramer (Fig. 1).^22^ A single RH heterodimer consists of the large, active site-containing subunit, HoxC, and the small subunit, HoxB, that harbors three iron–sulfur clusters (FeS) of so far unknown structure. Amino acid sequence analysis predicts the presence of three [4Fe–4S] clusters.^24^ This is in contrast to prototypical group 1 hydrogenases, which usually possess two [4Fe–4S] clusters and one [3Fe–4S] cluster,^2,4,17,27^ but is similar to the group 1 subclass of [NiFeSe]-hydrogenases.^28,29^

Though the molecular structure of regulatory hydrogenases is unknown, a number of biochemical and biophysical investigations by EPR,^20,21^ FTIR,^30^ Resonance Raman^31^ and X-ray Absorption Spectroscopy (XAS)^22,26^ have provided valuable information on the cofactor structure, composition and function of the RH from *R. eutropha*. Due to the high complexity of the system, *in vitro* studies were mainly carried out on a truncated version of the protein (designated as RH_stop_), which allows for both isolation by affinity chromatography and formation of a single heterodimer (HoxBC), the tertiary structure of which is similar to that of classical hydrogenases.^5,22^

The as-isolated (oxidized) RH_stop_ exhibits no EPR signals related to paramagnetic Ni or FeS clusters. In the most oxidized state, the [NiFe] active site resides in the EPR-silent, catalytically competent Ni-SI state,^20^ which contrasts the case of “standard” [NiFe]-hydrogenases that exhibit a superposition of paramagnetic states, known as Ni-A and Ni-B, reflecting oxidative modifications at the active site.^32–34^ The absence of the Ni-A and Ni-B states in RH, both of which require reductive reactivation, is fully consistent with the sensory character of the protein that needs to react immediately in the presence of H_2_.^22,24,35,36^ Incubation of RH with H_2_ in absence of artificial electron acceptors affords the paramagnetic Ni-C state characterized by a hydride ligand in the bridging position between the Ni^III^ and Fe^II^ ions.^20^ This state appears to be a thermodynamic “bottleneck”. Even prolonged incubation with H_2_ does not detectably yield the otherwise occurring most reduced Ni-R state(s), typically observed in standard hydrogenases. Ni-SI and Ni-C are therefore the only intermediates to accumulate in significant amounts in RH.^20^ The hydride ligand in Ni-C is photolabile at cryogenic temperatures due to its presumed translocation as a proton. This yields two well-defined paramagnetic forms, termed Ni-L_1_ and Ni-L_2_, where the Ni ion resides formally in the Ni^1+^ state.^28,37,38^

Paramagnetic states of the FeS clusters in RH so far have not been amenable to characterization by EPR spectroscopy. Therefore, the identity, electronic properties, and their possible role in the O_2_-tolerance of this enzyme have remained elusive. This prompted us to employ Mössbauer spectroscopy, which has proven to be a powerful tool for the characterization of the electronic structure of iron-containing cofactors in general and in [Fe]-,^1^ [FeFe]-,^39,40^ [NiFe]-^13,41–44^ and [NiFeSe]-hydrogenase, in particular.^45,46^ The study was complemented by EPR and FTIR spectroscopy. The Mössbauer spectra of RH can be explained by the presence of three [4Fe–4S] clusters, at least two of them with distinct redox properties. Strong reducing agents were employed to ascertain complete reduction of all three FeS clusters, which for the first time also afforded detectable EPR signals of the reduced FeS centers. Moreover, the low-spin Fe^II^ site in the [NiFe] center could be unambiguously identified, based on its unique properties which differ from those of other [NiFe] enzymes studied so far. The information obtained by Mössbauer spectroscopy was complemented by a ^57^Fe HYSCORE characterization of the [NiFe] center in the Ni-C state. Overall, the present study contains a comprehensive characterization of the metallocofactors in RH and sheds light onto their structural and electronic properties, which were so far enigmatic. On the basis of the present results the mechanism of the regulatory hydrogenase is revisited and discussed with regard to its physiological function – sensing of H_2_.

## Methodology

### Reagents

All chemicals were of analytical grade and used without further purification. ^57^Fe was purchased as metallic iron from Chemotrade Handelsgesellschaft, Düsseldorf (Germany) and subsequently dissolved in HCl to yield FeCl_3_ for preparing the fermentation media.

### Protein preparation

For the investigation of the RH by Mössbauer, EPR and FTIR spectroscopy, we have employed the so-called RH_stop_ protein, which represents a simplified version of the dimeric wild-type RH, comprising only one functional moiety of the dimer (Fig. 1).^22,26^ Because of the replacement of a 50 amino acid extension of the RH small subunit HoxB with a *Strep*-tagII affinity peptide, formation of the double-dimeric RH is prevented and instead the heterodimeric RH_stop_ protein can be obtained and further purified by affinity chromatography. The RH_stop_ derivative was purified from the transconjugant strain *R. eutropha* HF574 (pGE567),^19^ which was cultivated under hydrogenase-depressing conditions in the presence of ^57^FeCl_3_ as the sole source of iron. The RH was then purified by *Strep*-Tactin affinity chromatography as described previously.^19^ The elution fractions were pooled and the RH protein was subsequently concentrated to a final concentration of *ca.* 400 μM, suitable for spectroscopic analysis.

Reduction with H_2_ was performed by flushing the sample with hydrated H_2_ gas (99.99%, Air Liquide) for 60 minutes in an anaerobic glovebox (Coy). From these preparations, samples were obtained for parallel EPR, IR and Mössbauer experiments, respectively.

Reduction with reducing agents was carried out anaerobically through addition of either Ti^3+^ citrate or sodium dithionite (Sigma-Aldrich, Germany) to a final concentration of 10 mM. Sodium dithionite was freshly prepared in 100 mM Na_2_CO_3_/NaHCO_3_ buffer (pH 10). Ti^3+^ citrate was prepared by dissolving the necessary amount of TiCl_3_ (anhydrous, Sigma-Aldrich, Germany) in a solution containing ten-fold excess of tri-sodium citrate. The color of the solution turned immediately from violet to dark orange (final pH was 7.5). The Ti^3+^ stock concentration was 15 mM. In a first set of experiments, 1.5 equivalents of Ti^3+^ were added anaerobically to an RH sample and were allowed to react for 15 min, before dividing the sample in two aliquots and separate freezing for parallel examination by Mössbauer and EPR spectroscopy. In a second set of experiments, four more equivalents of Ti^3+^ were added to the previous Mössbauer sample to ensure complete reduction. No significant changes were observed in the spectra recorded with this sample, and it was therefore further used for field- and temperature-dependent Mössbauer spectroscopic studies.

### Mössbauer spectroscopy

Mössbauer spectra were recorded on an alternating constant acceleration spectrometer. The minimum experimental line width was 0.24 mm s^–1^ (full-width at half maximum). The sample temperature was maintained constant either in an Oxford Variox or an Oxford Mössbauer-Spectromag cryostat. The latter is a split-pair superconducting magnet system for applying fields of up to 8 Tesla to the samples that can be kept at temperatures in the range 1.5–250 K. The field at the sample is perpendicular to the γ beam. The ^57^Co/Rh source was positioned at room temperature inside the gap of the magnet in a reentrant bore tube at a distance of about 85 mm from the sample. The field is zero at this position. All isomer shifts are quoted relative to the centroid of the spectrum of metallic α-iron at 300 K. Mössbauer spectra were simulated employing the usual spin Hamiltonian formalism.^47^ Theoretical details are described in the ESI.†

### Electron paramagnetic resonance (EPR) spectroscopy

Continuous wave (cw) X-Band measurements were carried out using an X-band Bruker ESP 300E instrument (9.4 GHz, TE_012_ resonator) equipped with a helium flow cryostat (Oxford Instruments, ESR910) and an ITC 503 temperature controller. CW S-band (2–4 GHz, loop gap resonator) and CW Q-Band (34 GHz, TE_011_ resonator) measurements were carried out using a Bruker ESP 300 spectrometer equipped with an Oxford Instruments helium flow cryostat.

Pulse Q-Band experiments were carried out on a Bruker ELEXSYS E-580 FT EPR Q-band spectrometer equipped with a SuperQ-FT microwave bridge, a home-built slightly over-coupled cylindrical TE_011_ resonator, with a construction described by Reijerse *et al.*,^48^ and an Oxford CF935 Helium flow cryostat. The solid-state microwave amplifier in this bridge produces a power of 3 W at the resonator. Pulsed X-band EPR measurements were performed on a Bruker ELEXSYS E-580 X-band spectrometer with a SuperX-FT microwave bridge and a CF935 Oxford flow cryostat in the temperature range 10–20 K. An over-coupled Bruker ER 4118X-MD4-W1 dielectric ring ENDOR resonator was used for these experiments. The MW pulses were amplified by using an Applied Systems Engineering Traveling Wave Tube (TWT) amplifier (1 kW). Q-band and X-band HYSCORE spectra were recorded using the standard Bruker data acquisition software. W-band measurements were carried out on a Bruker ELEXSYS E-680 spectrometer, using the commercial W-band ENDOR probehead (Bruker).

Spin quantitation was carried out by using as a standard 1.0 mM Cu^2+^ chloride in water (1 mM Cu^2+^, 2 M NaClO_4_, 10 mM HCl), measured under non-saturating conditions. Simulations of the cw and pulse pseudo-modulated spectra, and HYSCORE spectra were done using home-written simulation programs implemented in the Kazan software (Dr Alexey Silakov, The Pennsylvania State University, and Prof. Boris Epel, University of Chicago) that employs MATLAB (Mathworks) as an interface. Details are described in the ESI† or in previous publications of our group.^49^

### Fourier transform infrared (FTIR) spectroscopy

Measurements were performed on a Bruker IFS 66v/s FTIR spectrometer with 2 cm^–1^ resolution. The detector was a photovoltaic mercury cadmium telluride (MCT) element. Room temperature measurements were done with a liquid cell that consists of two CaF_2_ windows (3.5 cm diameter) separated by a 0.1 mm Teflon spacer. The temperature was regulated by a thermostatic bath. The software for data recording consisted of the OPUS package (Bruker Optics). Analysis and further processing were performed with home-built routines written in MATLAB version 6.5 (Mathworks).

## Results

### The as-isolated, oxidized state of RH_stop_

**Fig. 2:**
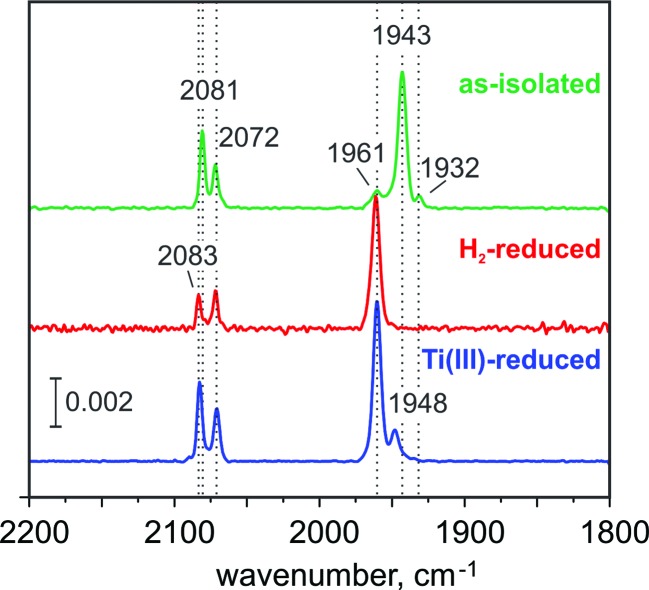
FTIR spectra of the RH from *R. eutropha* in its as-isolated (green trace), H_2_-reduced (red trace) and Ti^3+^ citrate-reduced (blue trace) states, which demonstrate the presence of 82% Ni-SI, 100% Ni-C and 85% Ni-C state, respectively. In the Ti^3+^-citrate treated sample the band at 1948 cm^–1^, may be correlated with a substoichiometric accumulation of Ni-R, an assignment that is however precluded, because the expected bands of the corresponding CN ligands of Ni-R are absent. The absence of any additional Ni signals in EPR, suggest that the 1948 cm^–1^ band corresponds either to a different conformation of Ni-C or to an EPR-silent state.

The FTIR spectrum of the as-isolated RH_stop_ (Fig. 2)^26,50^ exhibits three characteristic signals from the diatomic ligands at the Fe in the [NiFe] center that can be assigned to the Ni-SI state, with a strong band at 1943 cm^–1^ (corresponding to the stretching vibration of CO) and two weaker bands at 2081 cm^–1^, 2072 cm^–1^ (corresponding to the vibrationally coupled CN^–^ stretches).^2,51^ A small band at 1961 cm^–1^ coincides with the CO stretch of the Ni-C state, the concentration of which however, is presumably too low for the detection of the corresponding Ni-related EPR signals. Also the concentration of another minor component, causing a weak band at 1932 cm^–1^ appears to be undetectably low by EPR. The EPR spectrum of the as-isolated enzyme shows only faint signals at *g* = 4.23 and *g*_av_ ∼ 2.01, which we assign to adventitiously bound high-spin Fe^III^ (*S* = 5/2) and [3Fe–4S]^1+^ clusters (*S* = 1/2), respectively. Since in particular the [3Fe–4S]^1+^ clusters are present to only substoichiometric amounts (their spin quantification is in agreement with the abundance of only 0.3 clusters per protein dimer, as estimated below from the Mössbauer spectra), both EPR-active species appear to be oxidation-derived degradation products. This is a common observation for hydrogenases, because of the partial accessibility of O_2_ to the FeS clusters (the distal FeS cluster is particularly exposed to the protein surface).^52^

**Fig. 3:**
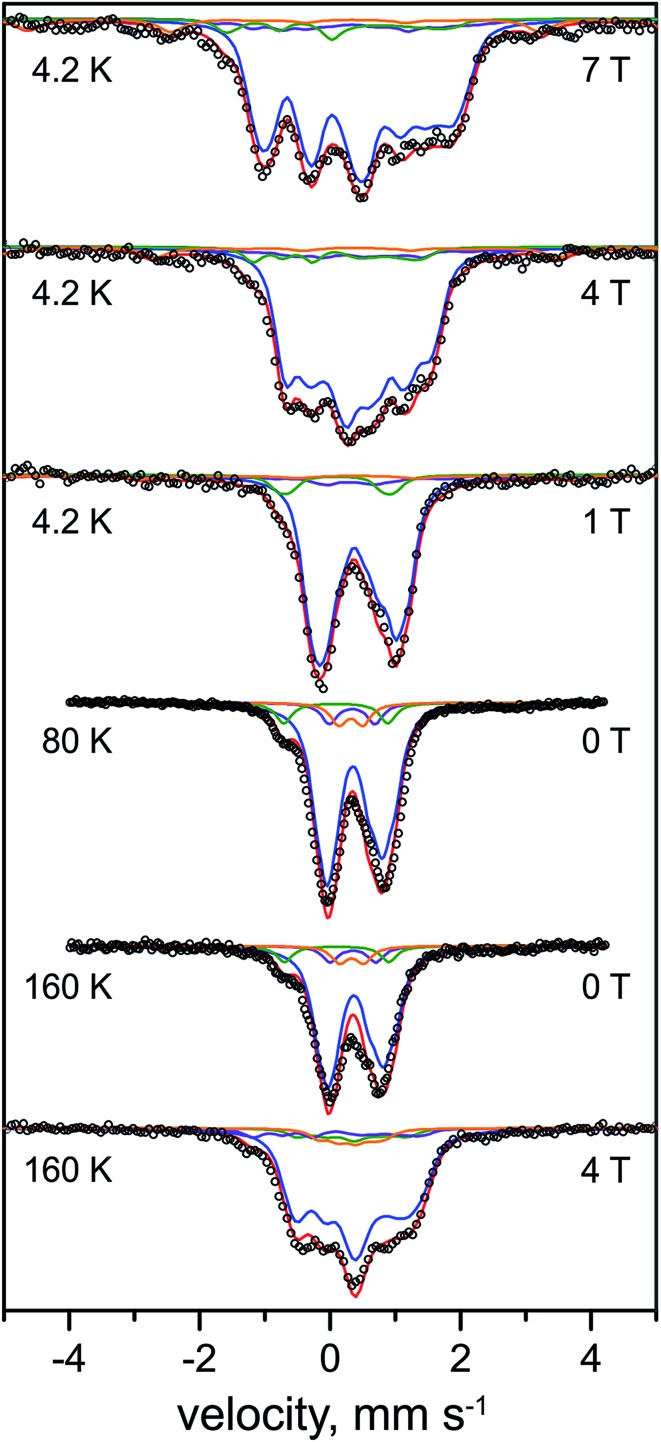
Mössbauer spectra of the as-purified form of ^57^Fe-enriched *R. eutropha* RH_stop_. Parameters for the simulations and experimental conditions are listed in Table 1. The magnetic field (in Tesla) was applied perpendicular to the γ-beam. Open circles represent the experimental data, solid lines represent the simulations: [4Fe–4S]^2+^ (blue line), [3Fe–4S]^+^ (purple line), low-spin Fe^II^ (green line), high-spin Fe^III^ (orange line), total (red line).

The Mössbauer spectra of the as-isolated ^57^Fe labeled RH_stop_ recorded at various magnetic fields are shown in Fig. 3. In these spectra all Fe containing species are detected irrespective of their redox and spin states. The dominant contribution (corresponding to ∼79% of the total iron content, blue lines) originates from a diamagnetic species with intermediately strong isomer shift and quadrupole coupling constants (Table 1). The values are typical of iron in the high spin state with quasi-tetrahedral sulfur coordination and mixed oxidation state (+2.5). Together with the diamagnetic behavior the features are characteristic for valence-delocalized [4Fe–4S]^2+^ clusters with four essentially indistinguishable Fe^2.5+^ sites and total spin *S* = 0.^53^ Other FeS clusters with Fe^2.5+^ sites would be either paramagnetic or have additional iron sites with different spectroscopically detectable oxidation states. Similar signals have been found in all [NiFe] hydrogenases studied by Mössbauer spectroscopy to date (Table S1†).^41–46^

**Table 1:** Mössbauer parameters of the Fe metallocofactors in the as-purified form of RH_stop_ ^*a*^

	Fe^II^ L.S.	[4Fe–4S]^2+^	[3Fe–4S]^+^	Fe^III^ H.S.
Rel. area (%)	7	79	7	7
*δ*, mm s^–1^	0.10	0.36, 0.38, 0.43, 0.44 (0.35, 0.37, 0.40, 0.41)	0.35, 0.35, 0.35	0.33
Δ*E*_Q_, mm s^–1^	1.60	0.50, 1.00, 1.30, 1.40 (0.50, 0.80, 0.90, 1.20)	–0.70, 0.70, 0.70	0.37
*η*		0.6, 0.6, 0.8, 0.8	0.2, 0, 0	
*A*_*x*_, *T*			–34, 10, 2.5	–15
*A*_*y*_, *T*			–34, 11, 2.5	–15
*A*_*z*_, *T*			–30, 11, 2.5	–15
*Γ*, mm s^–1^	0.30	0.30, 0.30, 0.30, 0.30	0.30, 0.30, 0.30	0.40

^*a*^Obtained from the simulations of the spectra at different applied fields perpendicular to the γ beam at 4.2 K. Isomer shifts (*δ*) and quadrupole splittings (Δ*E*_Q_) that are shown in parenthesis correspond to 160 K (these parameters were also used to simulate the spectrum at 80 K). *η* is the asymmetry parameter, *A*_i_ are the hyperfine tensor components and *Γ* is the line width. *g* = 2.0 was used for all the species. For the Fe^III^ high spin species *S* = 5/2, *D* = 1.0 cm^–1^ and *E*/*D* = 0.33 were assumed. The slow relaxation limit was assumed at 4.2 K and the fast relaxation limit was used at 160 and 80 K. Errors are estimated as follows: ± 0.01 mm s^–1^ (*δ*), ± 0.05 mm s^–1^ (Δ*E*_q_), ± 0.1 (*η*), ± 0.5 MHz (*A*).

A well-resolved shoulder observed in the zero-field spectra at *ca.* –0.8 mm s^–1^ can be ascribed to another, rather particular subspectrum with unusually low isomer shift and large quadrupole splitting (*δ* = 0.10 mm s^–1^, Δ*E*_Q_ = 1.60 mm s^–1^, green lines), accounting for ∼7% of the total Fe content. Because the isomer shift is clearly below the range known for physiological FeS sites (and their degradation products in proteins)^54–56^ this subspectrum can be associated with the low-spin Fe^II^ ion of the [NiFe] active site. As expected for the Ni-SI state, the Mössbauer spectrum reveals diamagnetic behavior, as can be seen, *e.g.*, from the field dependence of the low-energy line, yielding a shoulder throughout the series of spectra shown in Fig. 3.

Moreover, two weak paramagnetic components are present in the spectra, but they are of only minor intensity (∼7% of the total iron content each, Tables 1 and S1†). These can be attributed to the adventitiously bound high-spin Fe^III^ and a contamination with [3Fe–4S]^+^ clusters, respectively, which combined with their correspondingly weak EPR signals at *g* = 4.3 and *g*_av_ ∼2.01, suggest them to be oxidative degradation products (*vide supra*).

In summary, the relative intensities of the Mössbauer components correspond to a model with 13 genuine iron sites in RH_stop_, arising from 12 Fe in three [4Fe–4S] clusters and one iron in the [NiFe] active site (one Fe of 13 would correspond to 7.7% relative intensity, we observed ∼7% for the [NiFe] species). In practice minor decomposition of some cubanes can be inferred from the presence of nuisance Fe^III^ and [3Fe–4S] clusters, which changes the percentages slightly, revealing *ca.* 0.3 [3Fe–4S]^+^ clusters per heterodimer (see ESI†).

### The H_2_-reduced state of the RH_stop_

#### FTIR

The FTIR spectrum of the H_2_-reduced RH_stop_ exhibits three bands that are characteristic of the Ni-C state,^19^ with a CO stretching vibration at 1961 cm^–1^ and two CN-related stretches at 2083 and 2071 cm^–1^, respectively. This spectrum demonstrates that exposure to H_2_ leads to a well-defined and homogeneous reduction of the [NiFe] center to the Ni-C state (Fig. 2).

#### Mössbauer

**Fig. 4:**
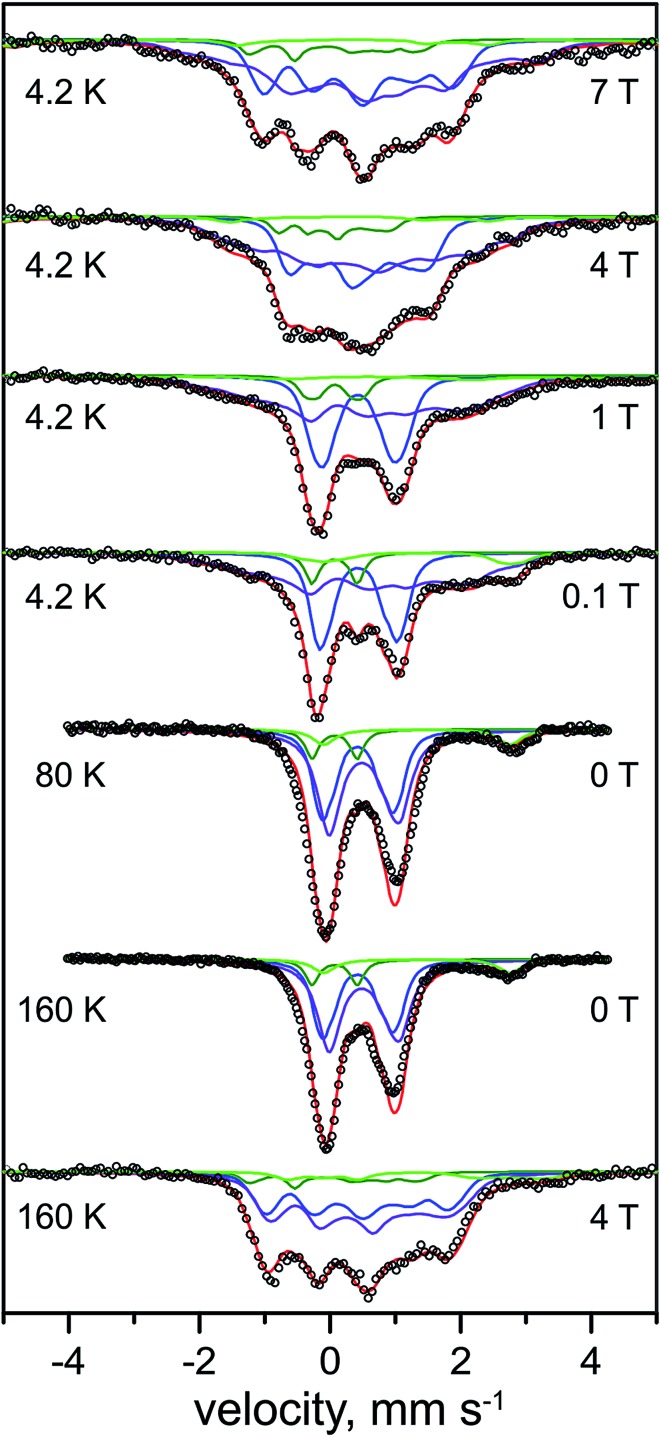
Mössbauer spectra of the H_2_-reduced form of ^57^Fe-enriched *R. eutropha* RH_stop_. Parameters for the simulations and experimental conditions are listed in Table 2. The magnetic field (in Tesla) was applied perpendicular to the γ-beam. Open circles are the experimental data, solid lines represent the simulations: total (red line), [4Fe–4S]^2+^ (blue line), [4Fe–4S]^+1^ (purple line), low-spin Fe^II^ (dark green line), N/O coordinated high-spin Fe^II^ (light green line).

The Mössbauer spectra of the H_2_-reduced RH_stop_ recorded at different magnetic fields and temperatures are shown in Fig. 4. The broad features found at low temperature and low fields indicate the presence of paramagnetic species, as expected for reduced [4Fe–4S] clusters. In accordance with previous Mössbauer results on standard [NiFe]- and [NiFeSe]-hydrogenases^41–46^ the spectra could be deconvoluted considering four components: (i) [4Fe–4S]^2+^ clusters with *S* = 0 ground state (∼36%, 1.2 clusters, blue lines), (ii) [4Fe–4S]^1+^ clusters with *S* = 1/2 ground state (∼49%, 1.6 clusters, purple lines), (iii) the low-spin Fe^II^ (*S* = 0) of the [NiFe] active site (∼7%, green lines), and (iv) high-spin Fe^II^ (*S* = 2) impurities (∼8%, light green lines), which, due to the high isomer shift, must have ‘hard’ N/O ligands and cannot represent a FeS-bound species.^57^

In support of this result, three different preparations of the H_2_-reduced RH_stop_ (Fig. S1†) exhibited rather consistent Fe speciation, with the respective yields of [4Fe–4S]^1+^ clusters amounting to ∼49–60% of the Fe content (*i.e.* 1.6–2.0 clusters). The results provide profound evidence for the reduction of tetranuclear iron–sulfur cofactors in the regulatory hydrogenase, a conclusion that has been drawn previously on the basis of UV/VIS and XAS spectroscopy, albeit invoking a different FeS cofactor composition.^19^ The intensity ratio of the Mössbauer subspectra demonstrates that maximally two of the three clusters in HoxB can be reduced by molecular H_2_.

Unique, model-free simulations of the complex magnetic Mössbauer spectra shown in Fig. 4 are virtually impossible in all details because of the large number of overlapping subspectra, which, even with the variety of field conditions and temperatures, cannot be fully disentangled. We were therefore aiming for 'generic' solutions, which allowed us to characterize the general types and numbers of different FeS clusters, and iron in the catalytic site. To this end, a set of Mössbauer parameters could be obtained (Table 2) by fine-tuning the literature values for iron–sulfur cofactors in standard [NiFe] and [NiFeSe] hydrogenases.^43,45,46,53^ These yielded the nice global simulation of the spectra shown in Fig. 4, but we cannot readily exclude ambiguities for each and every value of magnetic hyperfine coupling constants or individual isomer shifts and quadrupole splittings. However, the general assignments to oxidized and reduced [4Fe–4S] clusters are clear and the result excludes major deviations from the typical properties of cubane clusters.

**Table 2:** Mössbauer parameters of the Fe metallocofactors in H_2_-reduced RH_stop_ ^*a*^

	[4Fe–4S]^2+^	[4Fe–4S]^+1^	Fe^II^ L.S.	Fe^II^ H.S.
Rel. area (%)	36.0	49.0	8.0	7.0
*δ*, mm s^–1^	0.42, 0.43, 0.44, 0.45 (0.41, 0.42, 0.43, 0.44)	0.49, 0.62 (0.45, 0.53)	0.07	1.33
Δ*E*_Q_, mm s^–1^	0.80, 1.10, 1.20, 1.40 (0.70, 1.00, 1.10, 1.30)	1.32, 1.50 (0.90, 1.10)	0.69	2.85
*η*	0.6, 0.6, 0.8, 0.8	0.83, 0.11		
*A*_*x*_, *T*		–11.1, 18.8		–20
*A*_*y*_, *T*		–28.0, 4.0		–20
*A*_*z*_, *T*		–24.0, 10.4		–20
*Γ*, mm s^–1^	0.27, 0.27, 0.27, 0.27	0.5, 0.4	0.25	0.42

^*a*^This work, obtained from the simulations of the spectra at different applied fields perpendicular to the γ beam at 4.2 K. Isomer shifts (*δ*) and quadrupole splittings (Δ*E*_Q_) shown in parenthesis correspond to 160 K. *η* is the asymmetry parameter, *A*_i_ are the hyperfine tensor components and *Γ* is the line width. *g* = 2.0 was taken for all the species, otherwise stated. For the Fe^II^ high spin species *S* = 2, *D* = 10 cm^–1^ and *E*/*D* = 0.33 were assumed. The slow relaxation limit was assumed at 4.2 K and fast relaxation limit was used at 160 and 80 K. Errors are estimated as follows: ± 0.01 mm s^–1^ (*δ*) ± 0.05 mm s^–1^, (Δ*E*_Q_), ± 0.1 (*η*), ± 0.5 MHz (*A*).

**Table 3:** Electronic and magnetic parameters of the Ni-C spectrum employed for the simulation the X- and Q-band HYSCORE spectra of the ^57^Fe-labeled RH_stop_

	*x*	*y*	*z*	*α* ^*a*^	*β* ^*a*^	*γ* ^*a*^
*g*	2.197 ± 5 × 10^–3^	2.139 ± 5 × 10^–3^	2.015 ± 5 × 10^–3^			
*A* (MHz)	5.0 ± 0.5	1.1 ± 0.5	–0.5 ± 0.5	70 ± 5	20 ± 5	60 ± 5

^*a*^Principal angles of the hyperfine tensor with respect to the *g*-tensor axes.

The subspectrum of low-spin Fe^II^ in the catalytic center in the Ni-C state shows a similar low isomer shift (0.07 mm s^–1^) as found above for the oxidized Ni-SI state (0.10 mm s^–1^). This observation is in agreement with the active site iron persisting in its low-spin Fe^II^ state upon reduction of the RH with H_2_, which is supported by the diamagnetic behavior of the component in the magnetic Mössbauer spectra, revealing very small spin density on the Fe of the active site in the Ni-C state (*vide infra*). However, the quadrupole splitting (0.69 mm s^–1^) of the Fe^II^ ion in Ni-C is much lower than that in Ni-SI (1.60 mm s^–1^), revealing a significant change in the ligand environment.

A minor contribution from [3Fe–4S] clusters, as seen above by EPR for the oxidized protein, could not be unambiguously derived from the Mössbauer spectra of the reduced RH. Typical subspectra for the corresponding 1+ and 0 oxidation states of cubane trinuclear FeS centers with spins *S* = 1/2 and *S* = 2, respectively, were introduced in the simulations, but these did not significantly affect the quality of the fits or the distribution of the native FeS clusters (Fig. S2†), and therefore have not been further considered in our analysis. Presumably, magnetic broadening or heterogeneity in structure and environment of the non-physiological 3Fe clusters hampered their detection in the complex spectra of H_2_-reduced RH_stop_. Eventually, also reconstitution of [4Fe–4S] clusters from [3Fe–4S] species under reductive conditions in the presence of adventitious Fe^II^ ions cannot be completely ruled out as a rationale for the apparent absence of such signals in the spectra.^58^

### Reduction of RH_stop_ with strong reducing agents

#### FTIR

The FTIR spectrum of the Ti^3+^ citrate-reduced sample (Fig. 2) shows the typical Ni-C state signals.^26,50^ Even under these strongly reducing conditions (*E*_m_ =–510 mV at pH 7.5), the one-electron more reduced Ni-R state was not generated in detectable amounts, which is in agreement with previous observations.^23^ The Ni-R state, which is believed to be the direct outcome of the reaction of the Ni-SI state with H_2_,^59–62^ was accumulated neither with H_2_ (physiological) nor with more potent chemically reducing agents (non-physiological). Thus, Ni-C represents the only tractable reduced state of RH_stop_, which might be rationalized by the explicit redox equilibria of the RH cofactors (discussed below). A small band at 1948 cm^–1^, may indicate a substoichiometric generation of the Ni-R state.

#### Mössbauer

**Fig. 5:**
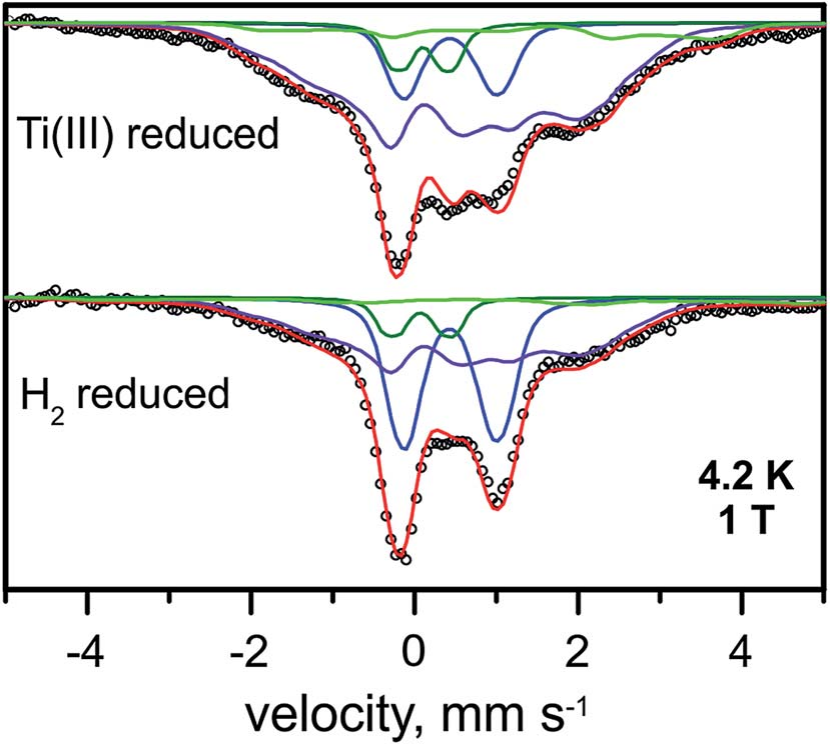
Mössbauer spectra of the Ti^3+^-citrate and the H_2_ reduced forms of the RH_stop_ recorded at 4.2 K and an external field of 1.0 T, applied perpendicular to the γ-beam. Parameters for the simulations and experimental conditions are listed in Table S3.† Open circles are the experimental data, [4Fe–4S]^2+^ (blue trace), [4Fe–4S]^1+^ (purple trace), Fe^II^ low spin of the [NiFe] site (green trace) and Fe^II^ high spin (light green trace).

The Mössbauer spectrum of the Ti^3+^ citrate-reduced RH_stop_ protein recorded at 4.2 K with 1 T applied magnetic field is shown in Fig. 5 (top), and a series of field- and temperature-dependent Mössbauer spectra is displayed in Fig. S7.† Employing the same approach and starting parameters as for the simulation of the spectra of the H_2_-reduced RH_stop_ (Tables 2 and S2†), analysis of the spectra of the Ti^3+^ citrate-reduced samples reveals almost full reduction of all three [4Fe–4S] clusters to the 1+ state (∼82% of the Fe in the sample, 2.67 clusters per protein). Similar results were obtained with samples reduced with sodium dithionite at pH 10 (data not shown). It should be noted that super-reduction of the cubanes to the [4Fe–4S]^0^ state, which would result in high isomer shift values and large quadrupole splittings exhibited by typical Fe^II^S_4_ sites, was not observed.^63–66^

The diamagnetic Mössbauer spectrum of the low-spin Fe^II^ ion in the [NiFe] center was identified reasonably well again, in particular in the spectra recorded at low temperature and small fields (Fig. 5 and S7†). In detail, the parameters could be taken the same as those determined for the H_2_-reduced samples (*δ* = 0.10 mm s^–1^, Δ*E*_Q_ = 0.69 mm s^–1^), which is supported by the FTIR spectra, demonstrating that the majority of the [NiFe] sites remains in the Ni-C state. Non-cluster-related high-spin Fe^II^ was also present as an impurity and amounted to ∼8%.

### EPR spectra of reduced RH_stop_

#### ‘Unsplit’ Ni-C signal of the H_2_-reduced RH_stop_

The X-band cw spectrum of the H_2_-reduced RH_stop_ shows at 5 K a single *S* = 1/2 signal with principal *g*-values (2.199, 2.140, 2.015) that are characteristic of the [NiFe] center in the Ni-C state (Fig. S2†), and which have been explained formally by adopting a configuration of a low-spin Fe^II^ (*S* = 0) and a Ni^III^ (*S* = 1/2) center, with the singly occupied orbital exhibiting mainly d_z_^2^ character.^20,21^ Remarkably, no indications from FeS resonances were found in the cw EPR spectra, although the majority of clusters were in the reduced state with spin *S* = 1/2 (see below). In addition, line broadening in the Ni-C spectra due to ^57^Fe hyperfine coupling was not observed for the ^57^Fe-enriched samples, which, according to the experimental line widths, renders the hyperfine coupling constants smaller than 15 MHz. Remarkably, the EPR spectra of the H_2_-reduced RH_stop_ did not show any evidence of magnetic interaction between the [NiFe] site and the ‘proximal’ [4Fe–4S]^1+^ cluster (known as ‘split’ Ni-C signal), although that cluster is proposed to be the first one to be reduced under physiological conditions.^43,51,57,58^ In contrast, the absence of a magnetic splitting in the Ni-C EPR signal supports the notion that the majority (if not all) of the proximal [4Fe–4S] clusters remain in their oxidized state upon H_2_ reduction under steady state conditions. Considering the fact that two FeS clusters reside in the 1+ state (Fig. 4) under these conditions, these have to be the medial and distal [4Fe–4S] clusters of the electron-transfer chain.

The numerical derivative of the pulse Q-band EPR spectrum shown in Fig. S4† discloses a ‘splitting’ in the *g*_*y*_ component of Ni-C. Because this splitting originates neither from ^57^Fe hyperfine interactions (spectrum of the ^56^Fe sample is identical) nor from magnetic interactions with the proximal [4Fe–4S]^1+^ cluster (splitting is field-independent), it is rather consistent with a conformational heterogeneity of Ni-C, which appeared to slightly vary between different sample preparations. This scenario is supported also by spectra obtained at higher microwave frequencies (*e.g.*, W-Band, Fig. S4†).

#### ^57^Fe hyperfine coupling in the Ni-C related EPR signal of H_2_-reduced RH_stop_

**Fig. 6:**
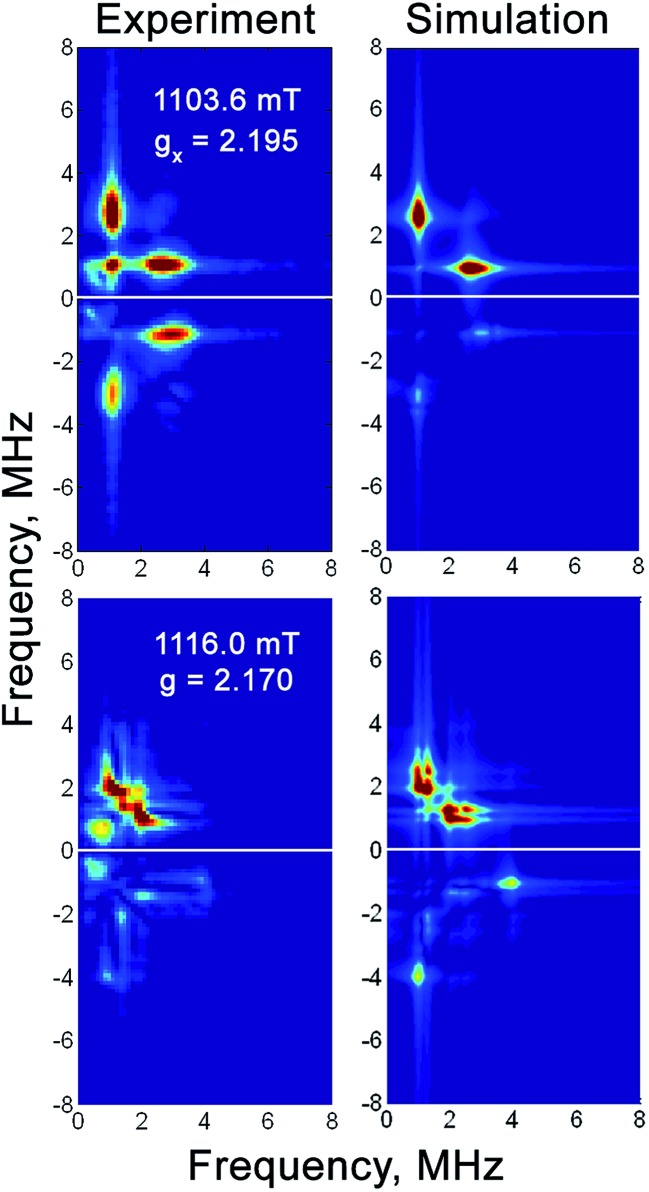
Q-band ^57^Fe HYSCORE spectra of the H_2_-reduced forms of ^57^Fe-enriched RH_stop_ at magnetic fields corresponding to two principal *g*-components of the Ni-C spectrum. Experimental conditions: mw frequency = 33.90 GHz, *T* = 20 K, π/2 = 40 ns, *τ* = 300 ns, shot repetition time = 1 ms. Simulation parameters are given in Table 3.

The weak magnetic hyperfine coupling of the low-spin Fe^II^ site (*S* = 0) in the Ni-C state, which is caused by (covalent) spin density delocalization from the Ni^III^ ion, could not be resolved by Mössbauer spectroscopy. Such weak interactions can be probed and studied by advanced pulsed EPR techniques.^32,67^ Indeed, 2- and 3-pulse ESEEM experiments at Q- and X-band frequencies showed intense modulations, originating from the electron spin interaction with the ^57^Fe nuclear spin (*I* = 1/2), which were absent in the ^56^Fe-labeled samples. Analyses of the orientation-selective HYSCORE spectra recorded at Q-band (Fig. 6 and S5†) and X-band (Fig. S6†) frequencies yielded principal values *A*_Fe_ = (5.0, 1.1, –0.5) (±0.5) MHz for the ^57^Fe hyperfine coupling tensor. Additional signals due to ^1^H and weakly coupled ^14^N nuclei from the protein backbone were also detected, in particular in the X-band HYSCORE spectra (Fig. S6†), but not further analyzed.

#### ‘Split’ Ni-C EPR signal in the chemically reduced RH_stop_

**Fig. 7:**
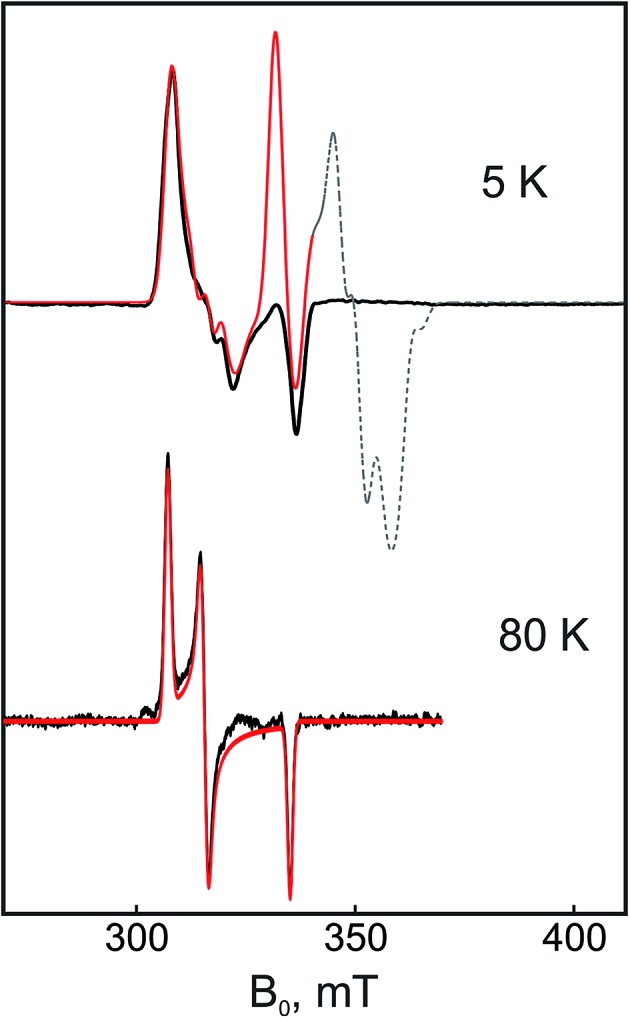
X-band CW EPR spectra of the dithionite-reduced RH_stop_ at two different temperatures, 5 K and 80 K, respectively. The experimental signals (black lines) and the simulations (red lines) correspond to the spin-coupled Ni-C sites since the contribution of the uncoupled Ni-C signal (‘unsplit’), estimated to be <30% in the spectra, has been subtracted from the spectra. The part of the simulated spectrum that corresponds to the [4Fe–4S]^1+^ cluster signals is depicted with a dashed line (this was experimentally not detectable presumably due to *g*-strain effects and relaxation enhancement caused by spin–spin interactions). Because of the fast electronic relaxation of the FeS cluster signals at 80 K, their signals are not detectable and thus the ‘fingerprint’ of their magnetic interaction on the [NiFe] is absent from the NiC-related EPR signals. Experimental conditions: mw frequency 9.47 GHz, modulation amplitude 0.7 mT, mw power 0.2 mW.

Incubation of the RH_stop_ with either Ti^3+^ citrate or dithionite resulted in the same Ni-C EPR spectrum without any discernable differences in the overall signal intensity. This is in contrast to canonical hydrogenases, in which the Ni-C signal diminishes upon prolonged activation with H_2_ as well as electrochemically at reduction potentials similar to those of the two strong reductants.^4,51^ The spectra of the Ti^3+^ citrate- and dithionite-treated RH_stop_ recorded at 40 K are shown in Fig. S8.† Lowering the temperature to ≤10 K led to the appearance of a 'split signal' (see also Fig. 7). This ‘splitting’ is indicative of magnetic interactions between the [NiFe] site and another paramagnetic species, which on the basis of its spatial localization (distance ≤ 14 Å) has to be the reduced proximal [4Fe–4S] cluster in the 1+ state. Similar ‘*interaction*’ spectra have been reported for all [NiFe]-hydrogenases studied so far, with the exception of the RH-type proteins.^20^ Hence, the ‘magnetic fingerprint’ found here is the first direct evidence of such a spin–spin interaction between adjacent metallocenters in a regulatory hydrogenase.

**Fig. 8:**
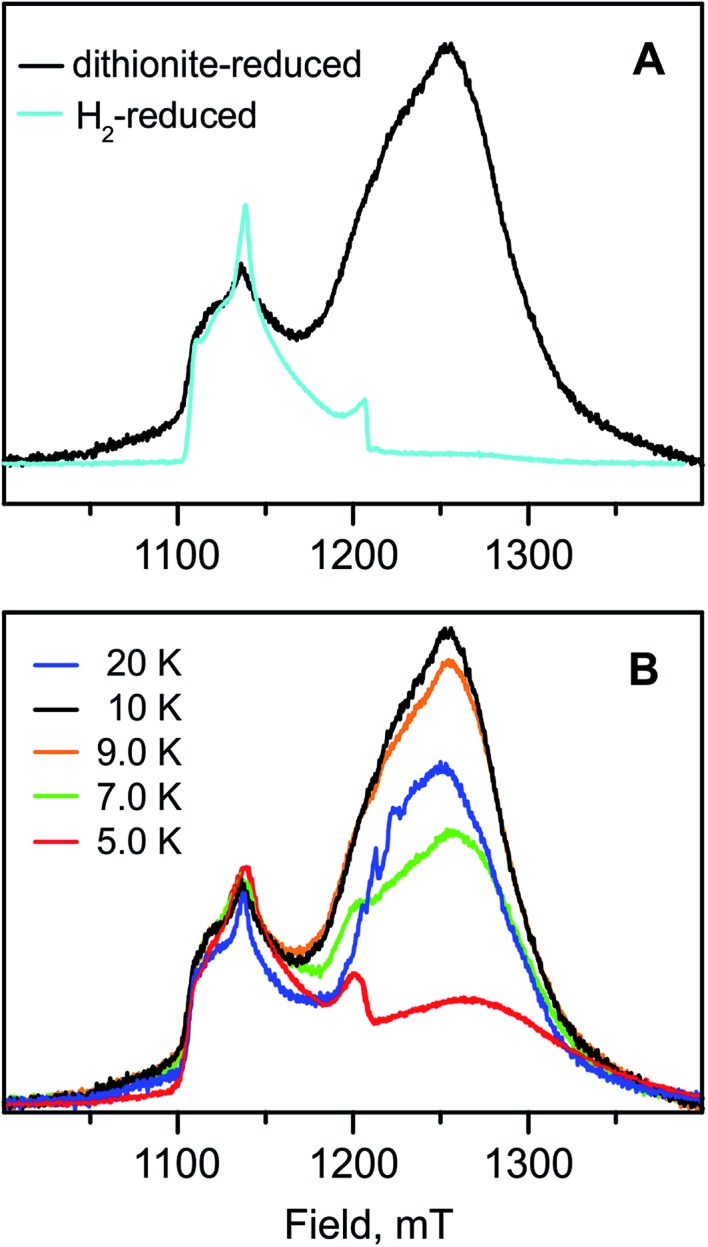
(A) Q-band field-swept 2-pulse echo-detected spectra of the H_2_-reduced (light blue trace) and sodium dithionite-reduced (black trace) forms of RH_stop_ (normalized to the Ni-C signal) recorded at 10 K. (B) Q-band field-swept 2-pulse echo-detected spectra of the sodium dithionite-reduced sample recorded at different temperatures. Experimental conditions: *τ* = 300 ns, π/2 = 16 ns, shot repetition time = 20 ms (H_2_-reduced). *τ* = 300 ns, π/2 = 12 ns, shot repetition time = 20 ms (dithionite-reduced).

The Mössbauer results suggest a certain heterogeneity with respect to the degree of reduction of the FeS clusters of the chemically reduced samples of RH_stop_, a fact that is reflected also in a mixture of 'split' and 'unsplit' Ni-C signals contributing to the EPR spectra. It is difficult to ascertain the precise amount of ‘unsplit’ Ni-C species in the spectra of the chemically reduced protein. However, by using the Ni-C signal of H_2_-reduced RH_stop_ as a reference for an almost pure ‘unsplit’ Ni-C spectrum, an upper threshold of less than 30% can be estimated for the fraction of magnetically uncoupled Ni-C in the dithionite-reduced sample (Fig. 7 and S8†). This estimation is in full agreement with the corresponding Mössbauer results on samples prepared under identical conditions, which demonstrated that 2.7 clusters (of three) are in the 1+ reduced state. With this presumption, the X- and Q-band Ni-C-related EPR spectra in the dithionite-reduced RH_stop_ could be well simulated to obtain a reasonable solution of the magnitude and orientation dependence of the electron spin–spin interaction between the participating paramagnetic centers. The spectral features can be reproduced by a simple three-spin model accounting for magnetic interaction between the [NiFe] site in the Ni-C state and the proximal [4Fe–4S]^1+^ cluster as well as between the proximal and the medial [4Fe–4S]^1+^ cluster. The interaction between the [NiFe] site and the medial cluster was assumed to be negligible and therefore not considered. The *g*-values of the [4Fe–4S]^1+^ clusters were chosen similar to those of other typical tetranuclear clusters, constrained, however, to reproduce the respective Q-band EPR spectra (Fig. 8, Table S2†). The interaction between the [NiFe] center and the proximal cluster was described by an anisotropic *J* tensor (*H*_electron spin–spin coupling_ = –*S*_1_·*J*·*S*_2_) with an isotropic component *J*_iso_ = 31.7 × 10^–4^ cm^–1^ (95 MHz) and an anisotropic component *J*_dip_ = [–60, 110, –50] MHz. The first is the trace of *J* and accounts for the through-bonds interaction of the paramagnetic centers, whereas the second part is traceless and accounts for through-space dipole interaction. Using the point-dipole approximation, *J*_dip_ is consistent with the [NiFe] site residing at a distance of *r* = 9.8 Å from the proximal [4Fe–4S]^1+^ cluster with spherical polar coordinates (*θ*, *φ*) = (80°, 80°) relative to the magnetic axes given by the two *g*-matrices, which were taken collinear.^68^ The interaction between the two [4Fe–4S]^1+^ centers was in good approximation considered to be isotropic with *J*_iso_ = 43.4 × 10^–4^ cm^–1^ (130 MHz). The signals of the [4Fe–4S]^1+^ clusters are not clearly detectable (presumably due to *g*-strain effects), which is similar to the case of standard O_2_-sensitive group 1 hydrogenases, but unlike [NiFeSe] hydrogenases.^68–70^

#### EPR spectra of the FeS clusters

Whereas the ‘split’ Ni-C EPR signal in chemically reduced RH_stop_ samples is readily observable by using conventional cw spectroscopy with field modulation, the signals of the reduced [4Fe–4S]^1+^ clusters are hardly detectable. Even measurements with dispersion-detection at temperatures as low as 2 K and high microwave power (providing rapid-passage conditions) were unsuccessful. Probably spin–spin interactions between the clusters, *g*-tensor anisotropy and *g*-strain effects broaden the signals beyond recognition. However, field-swept 2-pulse echo-detected Q-band spectra of H_2_- and dithionite-reduced RH_stop_ showed for the first time two distinct signals that can be assigned to Ni-C and the [4Fe–4S]^1+^ clusters (Fig. 8), respectively. The assignment is based on the typical low *g*-values of FeS clusters in conjunction with fast spin relaxation (the broad quasi-absorption signals are detectable only at lower temperatures, Fig. S3†). The intensity of the FeS subspectrum is significantly higher for the dithionite-reduced samples than that for the H_2_-reduced samples, given relative to the resolved Ni-C contribution, appearing at higher *g* values. This is in agreement with the Mössbauer results that demonstrated reduction of almost all three [4Fe–4S] clusters with dithionite. The region of the spectra in which the FeS clusters are expected could not be explored in the Ti^3+^-reduced samples, because the strong signal from residual reductant overlaps with the [4Fe–4S]^1+^ signals (Fig. S8†), hindering their detection and analysis.^21,24^

## Discussion

### The RH active site

The low-spin Fe^II^ site in the catalytic center of [NiFe] hydrogenases has not been discerned in most Mössbauer spectroscopic studies to date, because its spectrum is usually masked by the iron–sulfur cluster signals that impede site-resolution and characterization. In this study, however, we obtained well-resolved signals attributed to the corresponding low-spin Fe^II^ center in RH_stop_. This site exhibits a low isomer shift characteristic of low-spin Fe^II^ centers, because shielding of the ^57^Fe nucleus is less effective compared to the case of high-spin Fe^II^ centers.^71,72^ Furthermore, the quadrupole splitting parameter varies between the two different redox states. Upon transition from the oxidized Ni-SI to the one-electron more reduced Ni-C state, the quadrupole splitting Δ*E*_Q_ decreases from 1.60 mm s^–1^ to 0.69 mm s^–1^, reflecting significant changes in the ligand environment of the Fe^II^ ion, whereas the isomer shift remains almost invariant. Only minor differences in the isomer shift of the Fe^II^ ion were observed for the Ni-SI and Ni-C states, which suggest that there is no marked effect on the average bond lengths or the strength of the π-/σ-bond interaction with the diatomic Fe ligands. The quadrupole splitting, however, was significantly different for the two redox states, which might result from a change in the direct coordination environment of the Fe^II^. In fact, a vacant bridging position between the two heterometals has been proposed for the Ni-SI state, whereas in the Ni-C state, the presence of a bridging hydride between the Fe and Ni ions has been demonstrated.^20,37^ The observed decrease in the quadrupole splitting for the RH can therefore be correlated with the change in the Fe coordination number from Ni-SI (5-coordinate) to Ni-C (6-coordinate), the latter of which reflects a more symmetric environment. This significant difference of the quadrupole splitting between the Ni-SI and Ni-C states might not be a unique feature of H_2_-sensors and is expected to be observed also in other [NiFe]-hydrogenases. The unprecedented resolution of the low-spin Fe^II^ site (at least) in the as-isolated RH is presumably due to the almost stoichiometric presence of the Ni-SI state and the concomitant absence of any paramagnetic iron–sulfur cluster-related signals in the Mössbauer spectra ([3Fe–4S]^1+^, [3Fe–4S]^0^ or [4Fe–4S]^1+^).

X- and Q-band ^57^Fe HYSCORE experiments were carried out to characterize the electron spin density at the Fe center, from which detailed information about the electronic structure of the active site can be obtained. The anisotropic hyperfine tensor has an isotropic component of 1.9 MHz, which is two-fold larger than the ^57^Fe isotropic hyperfine coupling constants determined for the Ni-B state of the *D*. *vulgaris* hydrogenase (*A*_iso_ = 0.8 MHz)^33^ and the Ni-A state of the *D*. *gigas* enzyme (*A*_iso_ = 1.0 MHz).^73^ Because the isotropic hyperfine constant is proportional to the electron spin density at the nucleus, all data indicate a higher (although very small) electron spin density at the Fe center in the Ni-C state compared to the Ni-A and Ni-B states. Furthermore, the data are consistent with a low-spin Fe^II^ center in all states, with most of the spin density located at the Ni ion, as has been previously shown by ^61^Ni enrichment and subsequent determination of the respective ^61^Ni hyperfine tensor in the standard [NiFe] hydrogenase from *D*. *vulgaris*.^4,74,75^ These results are in full agreement with density functional theory calculations of the ^61^Ni hyperfine coupling constants.^76^

In the present as well as in previous studies of RH, the fully reduced Ni-R state could not be generated in significant amounts, neither by incubating the enzyme with H_2_ nor with strong reducing agents. In the FTIR spectra of the chemically reduced RH, a small intensity band at 1948 cm^–1^ is present and could represent the CO stretching vibration characteristic of the reduced Ni-R state. However, there is no evidence for the corresponding bands of the CN ligands, which for all reported Ni-R forms are typically shifted at least by 10 cm^–1^ to lower wavenumbers with respect to those of Ni-C.^51^ Nevertheless, we cannot exclude that Ni-R accumulated in small amounts, but the CN bands have been too low in intensity to be observed.

### The FeS clusters

Amino acid sequence analysis of the RH small subunit HoxB revealed 12 conserved cysteine residues to be the likely ligands of three predicted tetranuclear clusters.^24^ In previous studies, however, the iron–sulfur cofactors could not be successfully detected by EPR under any conditions.^20,21,23,50^ The unprecedented O_2_-tolerance (or rather insensitivity) of the RH protein and the limited spectroscopic accessibility of the iron–sulfur clusters invoked a series of proposals for the chemical nature and role of these metallocofactors in the function and O_2_-resistance of H_2_-sensing hydrogenases. In addition, previous XAS results suggested the presence of [2Fe–2S] clusters and an unusual [4Fe–3S–3O] cluster in the RH wild-type protein.^23,26^ However, these assumptions are not in line with our Mössbauer and EPR results, which neither showed signals attributable to [2Fe–2S] clusters^55,56,77^ nor to a [4Fe–4S] cluster with unusual oxygen ligation at one or more of the Fe sites. In the latter case, the more ionic oxygen ligands are expected to cause a greater electric field gradient asymmetry around that Fe site and result in a large quadrupole splitting, which, however, was not observed in our study. The Mössbauer and EPR results obtained for the RH_stop_ protein in combination with the presence of twelve conserved cysteine residues arranged in characteristic FeS cluster binding motifs are consistent with the presence of three [4Fe–4S] clusters in H_2_-sensing hydrogenases and afford for the first time the characterization of their electronic properties, which were found to be similar to those of classical tetranuclear cubanes. Without a crystal structure of the RH, however, unusual structures of any of the tetranuclear cubanes cannot be readily excluded.

In the as-isolated RH_stop_, all three clusters reside in their oxidized, diamagnetic [4Fe–4S]^2+^ form. Upon reduction with H_2_, approximately two (1.8) of the clusters are reduced to the 1+ state, whereas stronger (non-physiological) reducing agents, *i.e.* Ti^3+^ citrate and sodium dithionite, afford almost complete reduction of all three clusters (2.7). The expected EPR signals of the reduced [4Fe–4S]^1+^ clusters in both the H_2_ and dithionite/Ti^3+^ citrate-treated samples are hardly detectable, which may be a result of *g*-strain effects and the inter-cluster spin–spin interactions, as has been previously observed for group 1 hydrogenases.^43,68^

On the basis of the amino acid sequence, two of the three [4Fe–4S] clusters in the RH are coordinated by four cysteine-derived thiolate ligands, while the ‘distal’ cluster is coordinated by one His and three Cys residues. Thus, at least with respect to the first coordination sphere, the proximal and the distal clusters are expected to have similar electronic properties as their counterparts in the mostly O_2_-sensitive [NiFe]-hydrogenases. However, compared to other [NiFe]-hydrogenases, including the O_2_-tolerant membrane-bound enzymes, it is rather unusual that H_2_ incubation does not lead to detectable accumulation of the reduced state of the proximal [4Fe–4S] cluster. On the basis of our observations, the cluster remains largely oxidized under steady state conditions, which is also supported by the absence of a magnetic splitting in the Ni-C-derived EPR signal generated upon H_2_ incubation. The geometric arrangement of the clusters relative to the active site dictates that the proximal cluster is the first FeS cofactor to become reduced upon H_2_ oxidation. Thus, the present results may support a fast discharging of the proximal [4Fe–4S] center and rapid transfer of the electron(s) to the adjacent [4Fe–4S] centers (medial and then distal). The presence of a [4Fe–4S] cluster in the medial position suggests a rather streamlined electron transfer, *i.e.* the redox potentials of the [4Fe–4S] clusters and presumably that of the yet unknown cytoplasmic electron acceptor might be equalized in case of the H_2_ sensors. In most membrane-bound and periplasmic [NiFe] hydrogenases, a [3Fe–4S] cluster is found in the corresponding position.^4^

### Interaction between the [NiFe] active site and the FeS clusters

Although the FeS clusters of reduced RH_stop_ are essentially ‘silent’ in the cw EPR spectra, a spin coupling model of three spins *S*_i_ = 1/2 including the Ni center, the proximal and the medial clusters (both in their reduced 1+ forms) could be successfully employed to simulate the spectra of Ni-C in chemically (dithionite) reduced RH_stop_. In order to limit the number of parameters, the fourth paramagnetic species, the distal [4Fe–4S] cluster, was not included into the simulations.

In most hydrogenases studied so far, the long-range spin–spin interaction between the reduced proximal cluster and the [NiFe] center can be considered, in good approximation, to be isotropic. In the case of RH_stop_, however, the spin coupling tensor, *J*, is quite anisotropic as reflected in the Ni-C-related EPR spectra. The largest component of the anisotropic magnetic interaction tensor is 110 MHz and directed along the *y*-axis of the *g*-tensor of the Ni-C center, which is consistent with the absence of resolved splittings in the *g*_*x*_ and *g*_*z*_ components in the rhombic Ni-C spectrum. The isotropic component caused by the through-bond exchange interaction is found to be 95 MHz (assuming a traceless tensor for the anisotropic magnetic dipolar interaction).^78^ This value is comparable to the exchange coupling constant of *J*_iso_ = 120 MHz (40 × 10^–4^ cm^–1^) used for modeling the magnetic interaction between the paramagnetic active site in the Ni-C state and the proximal [4Fe–4S]^1+^ cluster in the standard hydrogenase of *D. gigas*.^68^ Recently, the exchange coupling constant between the active site in the Ni-B state and the superoxidized [4Fe–3S]^3+^ proximal cluster of the O_2_-tolerant hydrogenase I from *Aquifex (A.) aeolicus* was also found to lie in this range (*J*_iso_ = 107 MHz, 36 × 10^–4^ cm^–1^).^13^

The isotropic exchange coupling constant between the medial and the proximal clusters in RH_stop_ was determined to be at 130 MHz, which is noticeably larger compared to the corresponding value found in the case of *A. aeolicus* Hydrogenase I (21 MHz, 7 × 10^–4^ cm^–1^).^13^ This difference might provide an additional rationale for the difficulty to detect signals from the reduced [4Fe–4S] clusters in RH preparations due to a correspondingly increased sensitivity of the spectra for strain (distributions of different local electronic changes/interactions) and relaxation enhancement effects.

### Structure and function of H_2_-sensor hydrogenases

As described above, the RH harbors three [4Fe–4S] clusters and is in this respect structurally similar to the group 1 [NiFeSe] enzymes, which contain the same iron–sulfur cluster complement.^28,29^ The presence of a tetranuclear FeS cofactor in the medial position, which is usually occupied by a “high-potential” [3Fe–4S] center in most other hydrogenases, is consistent with the cytoplasmic localization of RH. In functional terms, the RH protein deviates from most other [NiFe(Se)] hydrogenases in some or all three of the following features: (i) it does not form the oxidized, inactive Ni-A and Ni-B states (ii) it hardly accumulates the most reduced Ni-R form upon reduction with H_2_, and (iii) H_2_ does not lead to detectable population of the proximal [4Fe–4S] cluster in the 1+ state. The first property is most likely related to the RH-specific narrow hydrophobic gas channel that restricts access of molecular oxygen to the active site,^18,19^ which apparently renders H_2_ sensors O_2_-insensitive rather than O_2_-tolerant. The second and third features are presumably relevant for the catalytic function underlying the H_2_-sensing mechanism of regulatory hydrogenases. Accumulation of the Ni-C state upon incubation of the RH with H_2_ and the apparent inability of RH to generate detectable amounts of the Ni-R state under steady-state conditions can be explained by one (or both) of the following thermodynamic considerations. First, the redox potential of the proton-coupled Ni-C to Ni-R transition might be more negative compared to that of standard hydrogenases. Second, the proximal [4Fe–4S] cluster may have an unusual low potential, which is supported by its difficult reducibility through H_2_. Assuming a redox potential that is appreciably more negative than that of the proton-coupled Ni-SI to Ni-C transition,^38^ the RH would be trapped in the Ni-C state under steady state conditions. The low-potential Ni-C to Ni-R transition may be promoted by alterations in the first and second coordination sphere of the RH active site, which might also result in lowering of the p*K*_a_ of the cysteine thiol-based proton present in the Ni-R state (*e.g.*, due to a more hydrophobic environment).^61^ One characteristic of the second coordination sphere of RH proteins is the presence of a glutamine residue instead of a histidine that is conserved in all other [NiFe]-hydrogenases. This glutamine residue might play a role in the redox properties of the active site but seems not to be involved in H_2_ sensing.^22^ The low redox potential of the proximal [4Fe–4S] cluster might also be caused by the immediate protein environment. In fact, a number of glycine residues in the vicinity of the proximal cluster, which are conserved only in sensory hydrogenases (Fig. S9†), are situated at the interface of the small and large subunits. It is difficult to predict how the presence of these conserved glycine residues may change the reduction potential, but presumably these may alter the local electrostatic environment.

Remarkably, a low redox potential of the proximal cluster would impede particularly the Ni-C to Ni-SI transition and thereby slowing down the catalytic activity of the enzyme. This is in-line with the low H_2_ turnover rate observed for H_2_ sensors, which, in turn, prevents energy loss and saves H_2_ for the energy-converting hydrogenases.^36^

## Summary and conclusions

The present study provides new insight into the nature and role of the metal cofactors in H_2_-sensing regulatory hydrogenases. Mössbauer spectroscopy allowed the identification of the low-spin Fe in the [NiFe] active site. As the Mössbauer parameters are sensitive to changes in the ligand field coordination and symmetry around the active site Fe ion, they can be used to probe structural and electronic changes otherwise inaccessible by other techniques. Only, the quadrupole splitting, but not the isomer shift, is markedly dependent on the redox state (Ni-SI and Ni-C) of the catalytic site. It is considerably smaller in the Ni-C state compared to that of the Ni-SI state, which correlates well with the iron being 6- and 5-fold coordinated respectively.

Mössbauer and EPR spectroscopy allowed for the first time the identification and characterization of three [4Fe–4S] clusters as constituents of the electron transfer relay in the RH. Their electronic properties are similar to those of conventional tetranuclear FeS centers in low-potential ferredoxins. The extent of their reduction is dependent on the chemical reductant employed, *i.e.* non-physiological, strong reducing agents led to reduction of essentially all three [4Fe–4S] clusters, while H_2_ afforded the reduction of only the medial and distal clusters. Upon H_2_ treatment, a magnetic ‘splitting’ in the EPR signal of the active site-related Ni-C state, which is typical for most [NiFe]-hydrogenases, was not observed for the RH. The splitting was detectable, however, when the enzyme was treated with dithionite and identified the proximal cluster as the last cofactor being reduced. This observation supports a low redox potential of the proximal cluster that promotes rapid transfer of electrons to the adjacent [4Fe–4S]^2+^ centers, which, on the basis of our data, are supposed to have higher redox potentials.

Concomitantly with the absence of the ‘split’ Ni-C EPR signal, also the Ni-R state as the most reduced catalytic intermediate is not accumulated upon “physiological” treatment of the RH with H_2_. Even in the presence of strong reducing agents, solely the Ni-C state is observed. Stabilization of the Ni-C state is presumably accomplished through appropriate adjustment of the redox potential of both the Ni-C-to-Ni-R transition (and the concomitant proton transfer) and the proximal [4Fe–4S] cluster. The potential of the latter seems to be too low to support an efficient redox transition from Ni-C to Ni-SI, which coincides with the low H_2_ turnover rates of H_2_-sensing regulatory hydrogenases. In summary, the present spectroscopic study unravels the structural basics that have been evolved to convert an energy-generating into a cytoplasmic, H_2_-sensing hydrogenase.
