# Supplementary material for: Cofactor composition and function of a H2-sensing regulatory hydrogenase as revealed by Mössbauer and EPR spectroscopy
Source: Chem Sci. 2015 May 26;6(8):4495–507. doi: 10.1039/c5sc01560j (PMC5665086; doi:10.1039/c5sc01560j)
Supplement: Supplementary file 1 [file SC-006-C5SC01560J-s001.pdf]

Electronic Supplementary Material (ESI) for Chemical Science.
This journal is © The Royal Society of Chemistry 2015

Cofactor composition and function of an H_2-sensing regulatory hydrogenase as revealed by Mössbauer and EPR spectroscopy

Supporting Information

Federico Roncaroli[a,b], Eckhard Bill*[,a], Bärbel Friedrich[c], Oliver Lenz[c,d], Wolfgang Lubitz*[,a] and Maria-Eirini Pandelia*[,e,a]

[a] *Max-Planck-Institut für Chemische Energiekonversion, Stiftstraße 34-36, 45470 Mülheim an der Ruhr, Germany*
[b] *Department of Condensed Matter Physics, Centro Atómico Constituyentes, Comisión Nacional de Energía Atómica (CNEA), Argentina*
[c] *Institut für Biologie / Mikrobiologie, Humboldt-Universität zu Berlin, Chausseestraße 117, 10115 Berlin, Germany*
[d] *Institut für Chemie, Technische Universität Berlin, Max-Volmer-Laboratorium, Straße des 17. Juni 135, 10623 Berlin, Germany*
[e] *The Pennsylvania State University, Department of Chemistry, State College PA 16802, USA*

*Corresponding authors: mxp65@psu.edu, wolfgang.lubitz@cec.mpg.de, eckhard.bill@cec.mpg.de

Mössbauer spectroscopy

The spin Hamiltonian for the ^{57}Fe nuclei with spin I in each magnetic component with electronic spin S, both in the ground and in excited state can be written as in equation [1,2] (Eq. 1).

$$H = \delta - \beta_N g_N \mathbf{B} \cdot \mathbf{I} + \frac{eQV_{zz}}{4I(2I+1)}\left[3I_z^2 - I(I+1) + \eta(I_x^2 - I_y^2\right] + \beta \mathbf{S} \cdot \mathbf{g} \cdot \mathbf{B} + D\left[\mathbf{S_z^2} - {}^1\!/_3\, S(S+1) + {}^E\!/_D\, (\mathbf{S_x^2} - \mathbf{S_y^2})\right] + \mathbf{S} \cdot \mathbf{A} \cdot \mathbf{I} \qquad \text{(Eq. 1)}$$

in which δ represents the isomer shift, the second term the nuclear Zeeman effect, the third term describes the quadrupole interaction, the fourth term the electronic Zeeman effect, the fifth term

the zero-field splitting (only relevant for species $S > ½$) and the last term the magnetic hyperfine interaction. The nuclear spin I takes values 1/2 and 3/2 for the ground and excited nuclear state, respectively. In the practical simulations using our own program MX (E. B.) the S operator in the expression of the hyperfine coupling could be replaced by its expectation values <S>, because fields > 20 mT have always been applied which are strong enough to safely decouple nuclear and electronic spin systems; eQ is the nuclear quadrupole moment, being 0 in the ground state; V_{zz} is the main component of the electric field gradient (EFG) tensor in the principal axis system. The coordinates of the EFG are conveniently chosen such that $|V_{xx}| \leq |V_{yy}| \leq |V_{zz}|$. η is the asymmetry parameter, defined as $\eta = (V_{xx} - V_{yy})/ V_{zz}$. **B** is the external applied field (vector). β is the Bohr magneton, β_N and g_N are the nuclear magneton and the nuclear g-factor, respectively. D and E/D are the zero-field splitting and the rhombicity parameters, respectively. The quadrupole interaction does not affect the ground state, but splits the nuclear excited state into two sublevels. As a result, in the absence of an external field, it produces a two-line Mössbauer absorption pattern with corresponding quadrupole splitting ΔE_Q given by Eq. 2 and centered at δ as referenced with respect to the zero velocity (x-axis)[1-3]:

$$\Delta E_Q = \frac{eQV_{zz}\sqrt{1+\frac{\eta^2}{3}}}{2} \qquad \text{(Eq. 2)}$$

ΔE_Q and η cannot be determined separately, and in order to accomplish that, measurements in the presence of an externally applied magnetic field B must be carried out. Because the field gradient tensor is traceless, i.e. $V_{xx} + V_{yy} + V_{zz} = 0$, the two parameters η and ΔE_Q are sufficient to define the quadrupole interaction.

Pulsed EPR spectroscopy

Simulations of the cw EPR spectra were performed by EasySpin- based programs, written in MATLAB (Mathworks).

Hyperfine sublevel correlation spectroscopy (HYSCORE) is basically a four pulse ESEEM technique whose pulse sequence is [4]: ((π/2) - τ - (π /2) - T_1 - τ - T_2 - (π /2)), and T_1 and T_2 time intervals. Modulation patterns (i.e. 2D plots of echo intensity vs d_x and d_y, which are the time increments the T_1 and T_2 intervals are varied) were measured at the magnetic field positions corresponding to the principal values of the g-tensor and some additional positions in between. From simulations of the field-dependent HYSCORE spectra, information about the full hyperfine tensors of the various magnetic nuclei was obtained. In total, 256 x 256 data points were acquired for d_x and d_y, T_1 and T_2 were 100 ns, and both time increments (d_x and d_y) were chosen to be 16 ns. The shot repetition time was 1000 μs, and a four-step phase cycle was used. The length of the mw pulses π/2 and π was optimized to the maximum available power. The τ value was optimized for each experiment in order to maximize the modulations due to the ^{57}Fe nuclei. The modulation patterns were baseline-corrected and multiplied with a Hamming window function. Zero filling was performed up to 1024 x 1024 points, and the array was subsequently Fourier transformed into a frequency domain to obtain magnitude contour spectra.

Analysis of the pulse EPR experimental data was carried out using home-written simulation programs in MATLAB 6.5. The spin Hamiltonian employed for obtaining the energy levels and their wave-functions for simulations of the HYSCORE spectra is essentially the same as the one described in Eq. 1, with the difference that there is the electronic Zeeman term and no nuclear excited states are present, i.e. there is no quadrupole interaction and no isomer shift.

$$H = -\beta_N g_N \mathbf{B} \cdot \mathbf{I} + \beta \mathbf{S} \cdot \mathbf{g} \cdot \mathbf{B} + \mathbf{S} \cdot \mathbf{A} \cdot \mathbf{I} \qquad \text{(Eq. 3)}$$

In Eq. 3, the first and second terms in the expression correspond to the electron and nuclear Zeeman interactions, respectively, with the external magnetic field **B**; the third term describes the hyperfine interaction defined by tensor **A**. The Ni-C state has an S = 1/2 electronic ground state. Since this work concerns investigation of the ^{57}Fe hyperfine interactions, the nuclear spin (I) in the ground state, I(^{57}Fe) = 1/2 and g_N(^{57}Fe) = 0.1806. In all calculations, the electron Zeeman interaction was assumed to be the dominant term, which is a good approximation for both the X- and Q-band frequencies. The orientation of the hyperfine interaction tensor was defined with respect to the principal axes of the electronic g tensor. In this study we use the 'y-convention' for rotation according to the Euler angles (α, β, γ). In this convention the first rotation is by angle α about the z-axis, the second is by angle β about the new y′-axis and the third is by angle γ about the new z′′-axes.

Table S1. Parameters obtained from the simulations of the Mössbauer spectra of the as-isolated form of RH from *R. eutropha*, comparison with other hydrogenases.

	RH from *R. eutropha* [a]				*D. gigas* [b] Standard hydrogenase	*D. baculatus* [c] [NiFeSe] hydrogenase
	$[4Fe\text{-}4S]^{2+}$	$[3Fe\text{-}4S]^{+}$	Fe^{II} L.S.	Fe^{III} H.S.	$[4Fe\text{-}4S]^{2+}$	$[4Fe\text{-}4S]^{2+}$
rel. Area.(%)	79.0	7.0	7.0	7.0		
δ, mm s^{-1}	0.36, 0.38, 0.43, 0.44 (0.35, 0.37, 0.40, 0.41)	0.35, 0.35, 0.35	0.10	0.33	0.41,0.44,0.43,0.44	0.38,0.42,0.42,0.46 (0.37, 0.40, 0.40, 0.42)
ΔE_Q, mm s^{-1}	0.50, 1.00, 1.30, 1.40 (0.50, 0.80, 0.90, 1.20)	-0.70, 0.70, 0.70	1.60	0.37	0.74, 1.00, 1.33, 1.32	0.78,1.05,1.25,1.40 (0.61, 0.93, 1.13, 1.37)
η	0.6, 0.6, 0.8, 0.8	0.2, 0, 0			0.65, 0.65, 0.80, 0.80	0.65, 0.65, 0.80, 0.80
A_x, T		-34, 10, 2.5		-15		
A_y,T		-34, 11, 2.5		-15		
A_z, T		-30, 11, 2.5		-15		
Γ, mm s^{-1}	0.30, 0.30, 0.30, 0.30	0.30, 0.30, 0.30	0.30	0.40		

[a]This work, obtained from the simulations of the spectra at different applied fields perpendicular to the γ beam at 4.2 K. Isomer shifts (δ) and quadrupole splittings (ΔE_Q) shown in parenthesis correspond to 160 K (these parameters were used to simulate the spectrum at 80 K). η is the asymmetry parameter, A_i are the hyperfine tensor components and Γ is the line width. g = 2.0 was for all the species. For the Fe^{III} high-spin species $S = 5/2$, D = 1.0 cm^{-1} and E/D = 0.33 were assumed. The slow relaxation limit was assumed at 4.2 K and the fast relaxation limit was used at 160 and 80 K. Errors are estimated as follows: ± 0.01 m s^{-1} (δ),± 0.05 m s^{-1} (ΔE_Q), ± 0.1 (η) and ± 0.5 MHz (A). [b] From references[5,6]. [c] From reference[7].

Assignments of the relative Mössbauer intensities to clusters contributions and other components for oxidized RH, as isolated:

Iron sites for a model with three genuine 4Fe-4S cluster per protein heterodimer:

$[4Fe\text{-}4S]^{2+}$	2×4 Fe,	from intact clusters
	$(1\text{-}\ \mathbf{x}) \times 4$ Fe,	from partially damaged cubanes
$[3Fe\text{-}4S]^{+}$	$\mathbf{x} \times 3$ Fe	
Fe^{II} L.S. , [NiFe]	1 Fe	
nuisance Fe^{III} H.S.	**y** Fe	

Total number of iron sites per protein dimer in average is then:

$$\Sigma = 2 \times 4 + (1\text{-}\ \mathbf{x}) \times 4 + \mathbf{x} \times 3 \text{ Fe} + 1 + \mathbf{y} \text{ Fe}$$

With the experimental result that the 3Fe cluster account for 7% of the total iron content

$$3\,\mathbf{x} = 0.07 \times \Sigma$$

and nuisance iron accounts for 7% of the total iron content as well,

$$\mathbf{y} = 0.07 \times \Sigma$$

we arrive at a total number of iron sites:

Σ = 13,64 Fe.

Conclusions:

One Fe in the [NiFe] center should accounts for 1 Fe / 13.64 Fe = 0.073 = **7.3 %** of the total Mössbauer intensity (exp. 7 %).

The number of 3Fe4S clusters (7% rel. intensity) is $\mathbf{x} = 0.07 \times \Sigma / 3 = 0.07 \times 13.64 / 3 = 0.32$ clusters per protein heterodimer.

Table S2. Parameters obtained from the simulations of the Mössbauer spectra of the H_2-reduced form of the RH from *R. eutropha*, comparison with other hydrogenases

	RH from *R. eutropha* [a]				*B. stearothermophilus* reduced ferredoxin [b]	*D. baculatus* [NiFeSe] hydrogenase [c]	*D.desulfuricans* [NiFe] hydrogenase [d]
	$[4Fe\text{-}4S]^{2+}$	$[4Fe\text{-}4S]^{+}$	Fe^{II} L.S.	Fe^{II} H.S.	$[4Fe\text{-}4S]^{+}$	$[4Fe\text{-}4S]^{+}$	$[4Fe\text{-}4S]^{+}$
δ, mm s^{-1}	0.42, 0.43, 0.44, 0.45	0.49, 0.62	0.07	1.33	0.50, 0.58	0.42, 0.50, 0.55, 0.55	0.53, 0.58
	(0.41,0.42,0.43,0.44)	(0.45, 0.53)					
ΔE_Q, mm s^{-1}	0.80, 1.10, 1.20, 1.40	1.32, 1.50	0.69	2.85	1.32,1.89	-1.2, 1.0, 1.3, 1.7	1.29, 1.88
	(0.70,1.00,1.10,1.30)	(0.90, 1.10)					
η	0.6, 0.6, 0.8, 0.8	0.83, 0.11			0.78, 0.32	1.3, 0.3, 0.2, 1.2	0.78, 0.32
A_x, T		-11.1, 18.8		-20	-23.0, 19.2	-24.0, 3.0, -18.0, 8.3	12.3, -8.0
A_y,T		-28.0, 4.0		-20	-23.6, 9.8	-21.0, 3.0,-22.0, 8.3	14.9, -3.3
A_z, T		-24.0,10.4		-20	-20.0, 6.3	-6.0, 3.0, -25.0, 8.3	15.2, -3.1
Γ, mmm s^{-1}	0.27, 0.27, 0.27, 0.27	0.5, 0.4	0.25	0.42	0.27, 0.27		0.45, 0.40

[a] This work, obtained from the simulations of the spectra at different applied fields perpendicular to the γ beam at 4.2 K. Isomer shifts (δ) and quadrupole splittings (ΔE_Q) shown in parenthesis correspond to 160 K (used to simulate spectrum at 80 K). η is the asymmetry parameter, A_i are the hyperfine tensor components and Γ is the line width. g = 2.0 was taken for all the species. For the Fe^{II} high spin species S = 2, D = 10 cm^{-1} and E/D = 0.33 were assumed. Errors are estimated as follows: ± 0.01 m s^{-1} (δ),± 0.05 m s^{-1} (ΔE_Q), ± 0.1 (η) and ± 0.5 MHz (A). (The slow relaxation limit was used at 4.2 K and the fast relaxation limit was used at 160 and 80 K. [b] From reference[8]. [c] From references [5,6]. [d] From reference[7].

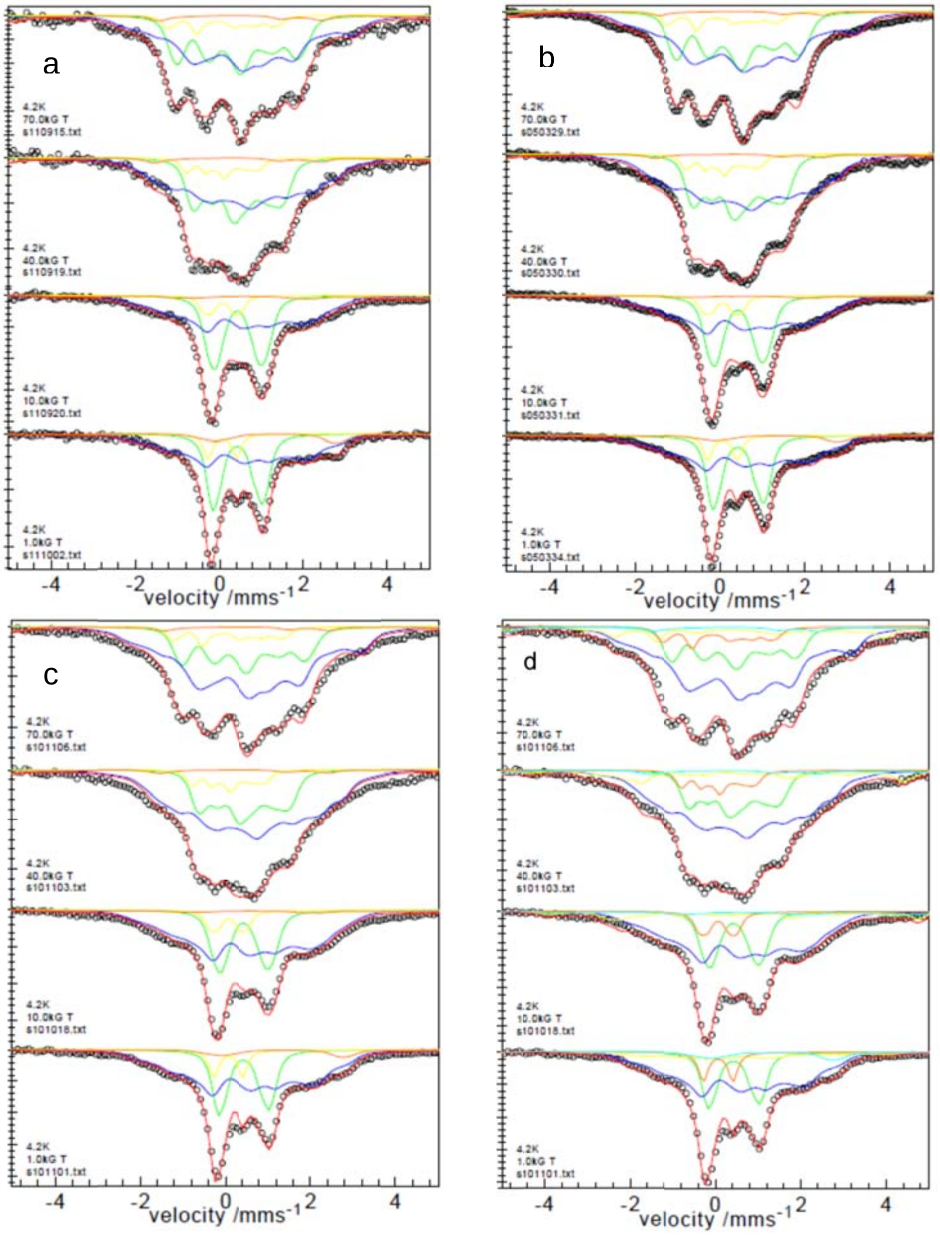

Figure S1. Mössbauer spectra of three different preparations of the H_2-reduced form of the RH from *R. eutropha* at 4.2 K and different applied fields. The parameters listed in Table S2 were used in all simulations. The samples exhibited slightly different speciations: **a**) $[4Fe\text{-}4S]^{2+}$ 36 %, $[4Fe\text{-}4S]^{+}$ 49 %, Fe^{II} low-spin (active site) 8.0 % and Fe^{II} high-spin 7.0 %. **b**) $[4Fe\text{-}4S]^{2+}$ 35 %, $[4Fe\text{-}4S]^{+}$ 52 %, Fe^{II} low-spin (active site) 8.0 % and Fe^{II} high-spin 5.0 %. **c**) $[4Fe\text{-}4S]^{2+}$ 27 %, $[4Fe\text{-}4S]^{+}$ 60 %, Fe^{II} low-spin (active site) 8.0 % and Fe^{II} high-spin 5.0 %. **d**) like c), but 10 % of a $[3Fe\text{-}4S]^{0}$ cluster was considered, parameters: g = 2.0, D = -2.0 cm^{-1}, E/D = 0.2, δ =

(0.46,0.46,0.32) mm^{-1}, Γ = 0.3 mm^{-1}, ΔE_Q = (1.47, 1.47, -0.52) mm s^{-1}, η = (0.40, 0.40, -1.0), A_x= (-14.9, -14.9, 9.9) T, A_y = (-14.9,-14.9,11.6) T, A_z = (-11.6,-11.6,12.6) T. The integration of the other species is: 22 % $[4Fe\text{-}4S]^{2+}$, 55 % $[4Fe\text{-}4S]^{+}$, 8 % Fe^{II} low spin and 5 % Fe^{II} high-spin.

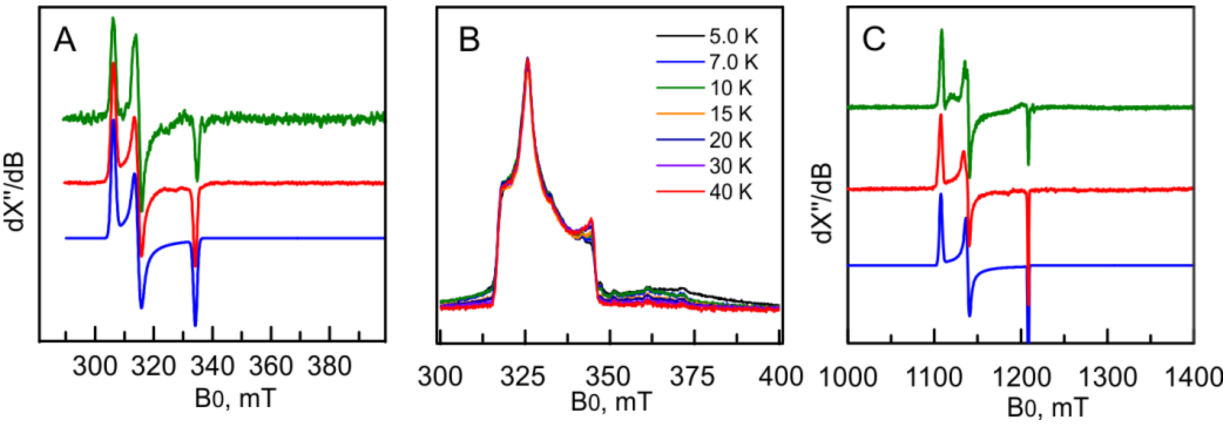

Figure S2. A) X-band EPR spectra of the H_2-reduced form of the RH *from R. eutropha*. Green: 1.0 mT pseudo-modulated first derivative of the field-swept 2-pulse echo-detected spectrum, T = 5 K, τ = 300 ns, $\pi/2$ = 6 ns, shot repetition time (SRT) = 50 ms. Red: cw EPR spectrum, T = 5 K, mw frequency = 9.43 GHz, mw power = 0.20 mW, modulation amplitude = 0.5 mT. Blue: simulation: g = (2.199, 2.140, 2.015), linewidth 1.5 mT, *g*-strain = (0.0068, 0.0106, 0.0). **B**) X-band field-swept 2-pulse echo-detected spectra at different temperatures, τ = 300 ns, $\pi/2$ = 6 ns. **C**) Q-band EPR spectra. Green: pseudo-modulated first derivative of the field-swept 2-pulse echo-detected spectrum, 1.5 mT pseudo-modulated, T = 5 K, τ = 300 ns, $\pi/2$ = 12 ns, SRT = 30 ms, frequency = 34.03 GHz. Red: cw spectrum, T = 40 K, mw. frequency = 34.10 GHz, mw power = 0.12 mW, modulation amplitude = 0.5 mT. Blue: simulation: $\boldsymbol{g}$ = (2.200, 2.140, 2.016), linewidth 1.4 mT, *g*-strain = (0.007, 0.007, 0.0).

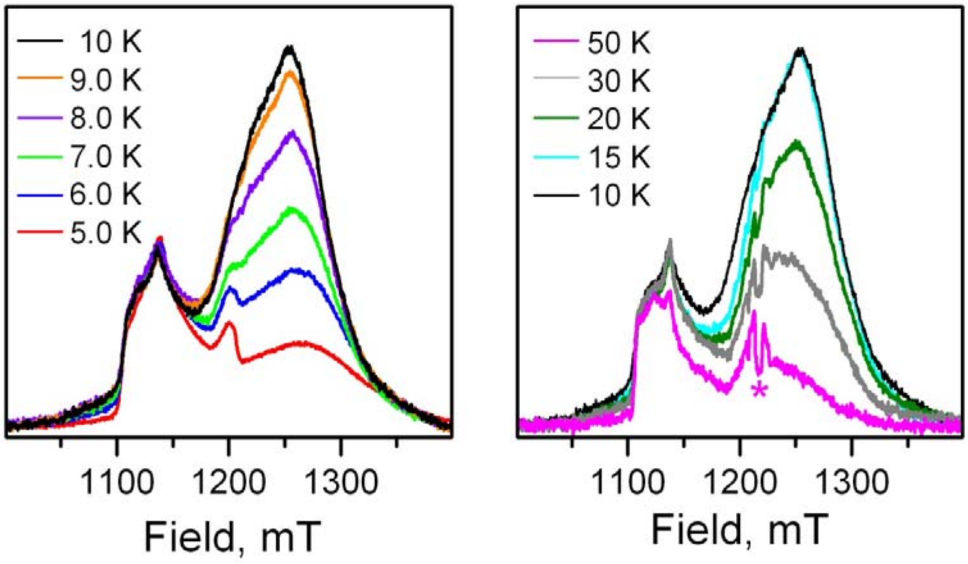

Figure S3. Q-band field-swept 2-pulse echo-detected spectra of the sodium dithionite reduced *Re* RH_{stop} at different temperatures, τ = 300 ns, $\pi/2$ = 12 ns. The signals marked with an asterisk are due to Mn^{2+} impurities.

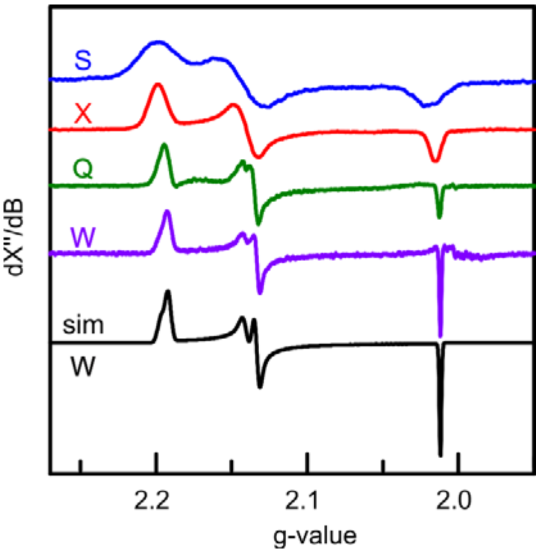

Figure S4. EPR spectra of the H_2-reduced RH from *R. eutropha*. **S**-band: cw EPR spectrum, T = 5 K, mw frequency 3.56 GHz, mw power 1.87 x 10^{-3} mW, modulation amplitude 0.5 mT. **X**-band: cw EPR spectrum, T = 5 K, mw frequency = 9.43 GHz, mw power = 0.2 mW, modulation amplitude = 0.5 mT. **Q**-band: 1.5 mT pseudo-modulated derivative of the 2-pulse field-swept echo-detected EPR spectrum, T = 5 K, mw frequency = 34.04 GHz, τ = 300 ns, $\pi/2$ =12 ns, shot repetition time (SRT)= 30 ms. **W**-band: 7.0 mT pseudo-modulated derivative of the 2-pulse field-swept echo-detected EPR spectrum, T = 10 K, mw frequency = 94.05 GHz, τ = 300 ns, π /2 = 20 ns, shot repetition time = 4 ms. Simulation parameters (W-band): two non-interacting spin systems with g_1 = (2.196, 2.140, 2.012), linewidth$_1$ = 2.7 mT, g-strain$_1$ = (0.0050, 0.0050, 0) (37.5%); g_2 = (2.192, 2.133, 2.012), linewidth$_2$ = 2.7 mT, g-strain$_2$ = (0.0040, 0.0033, 0), (62.5%).

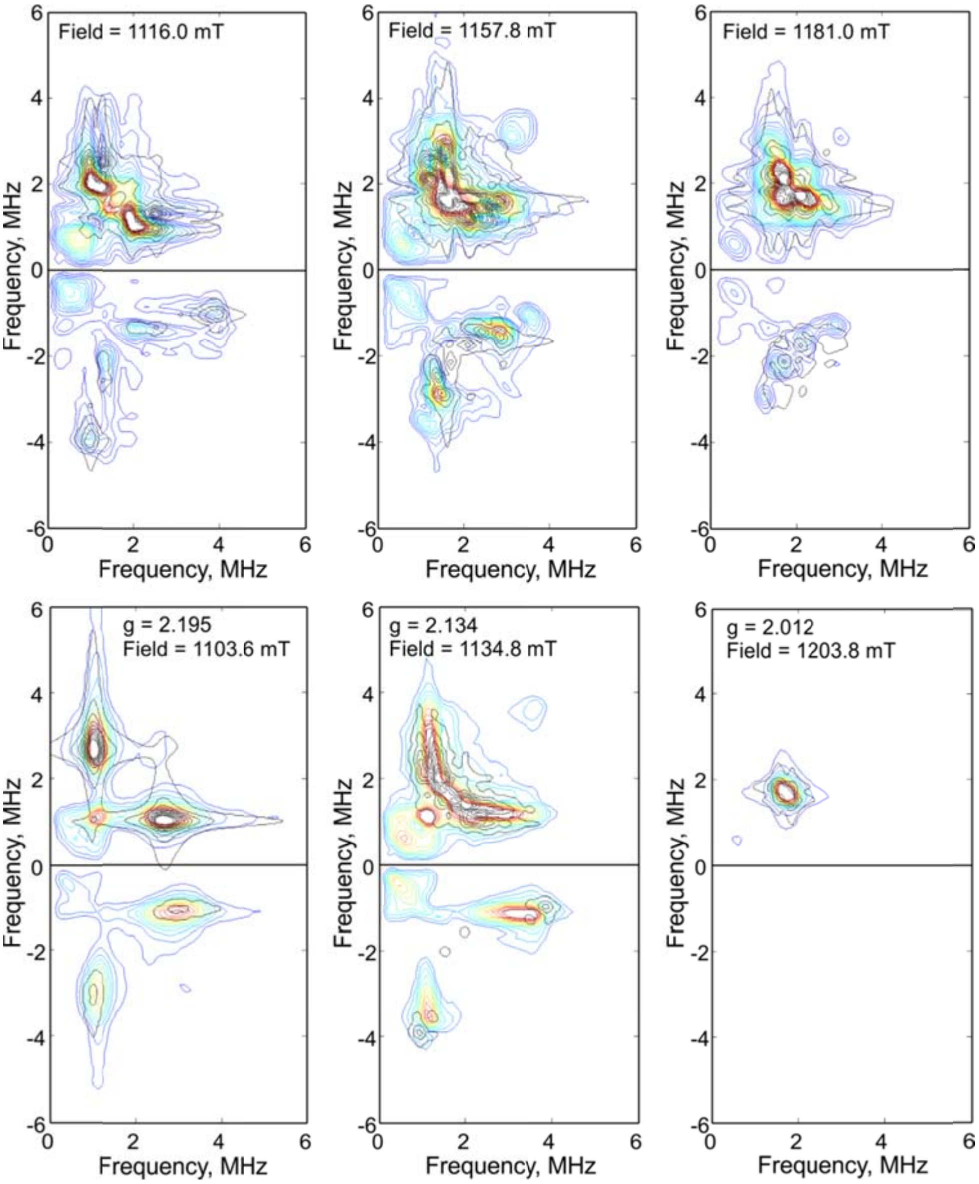

Figure S5. Q-band ^{57}Fe HYSCORE spectra of the H_2-reduced form of the RH from *R. eutropha.* Mw frequency = 33.90 GHz, T = 20 K, $\pi/2$ = 40 ns, τ = 300 ns, SRT = 1 ms. Simulations overlaid in black, g = (2.197, 2.139, 2.015) (± 5 x 10^{-3}), A = (5.0, 1.1, -0.5) (± 0.5) MHz, Euler angles (70, 20, 60) (± 5).

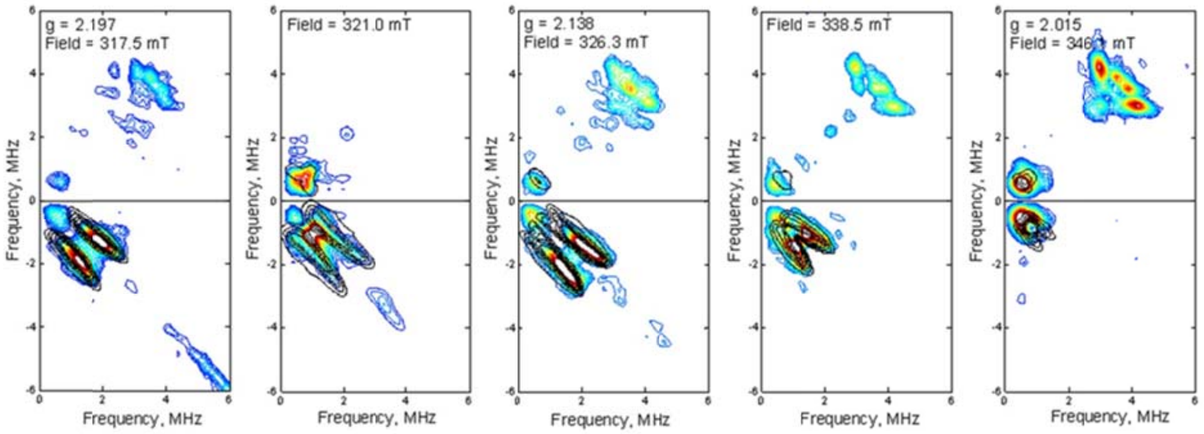

Figure S6. X-band HYSCORE spectra of the H_2-reduced form of the RH from *R.eutropha.* Mw frequency = 9.76 GHz, T = 20 K, $\pi/2$ = 8 ns, τ= 140 ns, shot repetition time = 1 ms. The numerical simulations for the ^{57}Fe nucleus are overlaid in black, g = (2.197, 2.139, 2.015) (± 5 x 10^{-3}), A = (5.0, 1.1, -0.5) (± 0.5) MHz, Euler angles (70, 20, 60) (± 5). For the spectrum at 321 mT a τ = 290 ns was used.

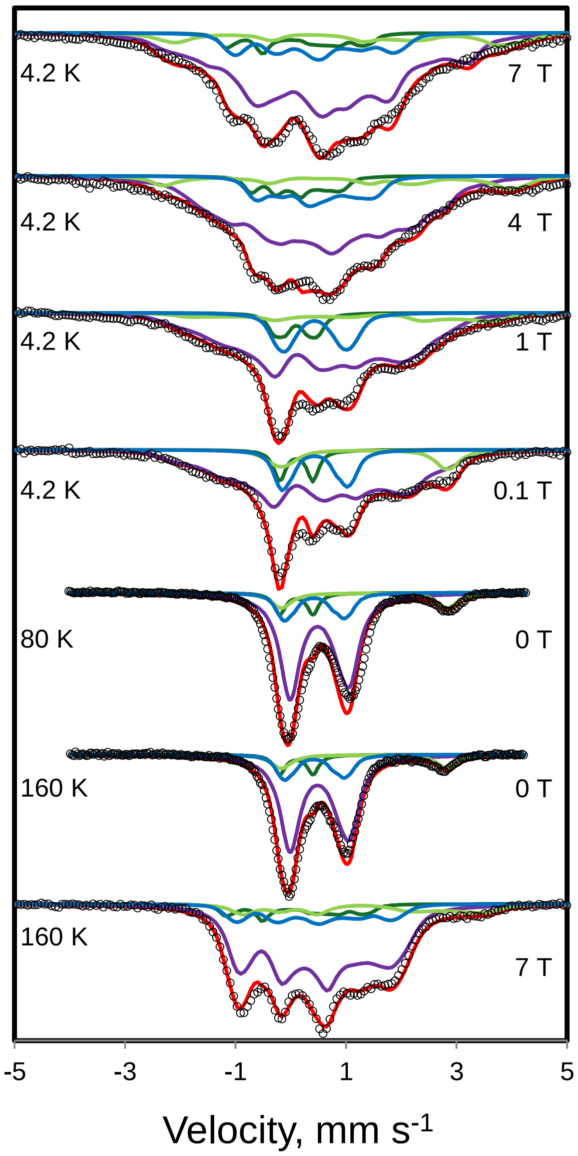

Figure S7 Mössbauer spectra of the Ti^{3+} citrate-reduced RH_{stop} from *R. eutropha*. The parameters employed in the simulations and experimental conditions are listed in Table 2 in the main text of the manuscript. Open circles are the experimental data, total simulation (red trace), $[4Fe\text{-}4S]^{2+}$ (blue trace) $[4Fe\text{-}4S]^{+}$ (purple trace), Fe^{II} low-spin (green trace), Fe^{II} high-spin (light green trace).

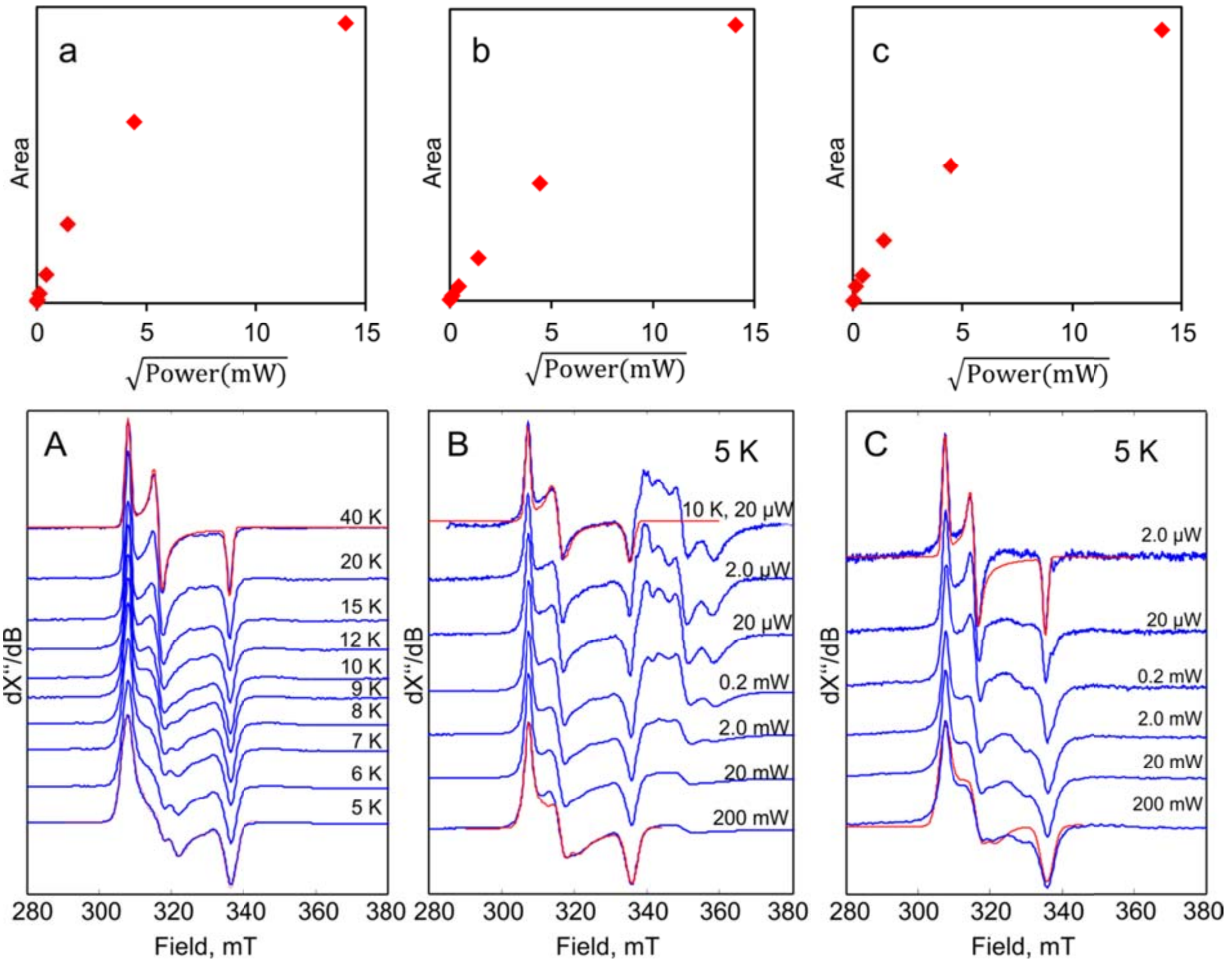

Figure S8 X-band continuous microwave power saturation of the Ni-C signals obtained upon reduction with: a) sodium dithionite, b) Ti^{3+}-citrate, c) H_2, all measured at 5 K. The area was obtained by double integrating the simulated cw spectra. **A)** X-band cw spectra of the sodium dithionite reduced RH at different temperatures, mw. frequency 9.47 GHz, modulation amplitude 0.7 mT, mw power 0.2 mW (except at 5 K, 2.0 mW). Parameters for the simulation of the spectrum at 40 K: g = (2.197, 2.139, 2.014), line width = 1.7 mT, g-strain = (0, 0.0087, 0). **B)** X-band cw spectra of the Ti^{3+}-citrate reduced RH at different mw powers. T = 5.0 K (otherwise stated), mw frequency 9.46 GHz, modulation amplitude 0.7 mT. **C)** X-band cw spectra of the H_2-reduced RH at different mw powers. T = 5.0 K, mw frequency 9.46 GHz, modulation amplitude 0.7 mT. In blue: experimental data, in red: simulation. Similar *g*-values were employed for the simulation of the spectra at lower temperatures, albeit with slightly different linewidths.

Table S3. Principal *g*-values of the metallocofactors used to simulate the magnetically coupled EPR spectra shown in Figure 7 (5 K) in the main text. A three spin model with three S=1/2 systems was considered (e.g. [NiFe] site, proximal $[4Fe\text{-}4S]^{1+}$ and distal $[4Fe\text{-}4S]^{1+}$ clusters). The exchange interaction between the [Ni-Fe] site in the Ni-C state and the proximal [4Fe-4S] cluster is anisotropic with J_{iso} = 95 MHz and J_{dip} = [-60 110 -50] MHz. The spin-spin interaction between the proximal [4Fe-4S] cluster and the medial one was considered in good approximation isotropic and equal to 130 MHz.

	g_1	g_2	g_3
**Ni-C**	2.196	2.136	2.011
**$[4Fe\text{-}4S]_{pr}$**	2.045	1.94	1.90
**$[4Fe\text{-}4S]_{med}$**	2.035	1.94	1.87

Table S4. Parameters obtained from the simulations of the Mössbauer spectra of the Ti^{3+} citrate-reduced RH from *R.eutropha.* [a]

	$[4Fe\text{-}4S]^{2+}$	$[4Fe\text{-}4S]^{+}$	Fe^{II} L.S.	Fe^{II} H.S.
rel. Area.(%)	15.0	67.0	8.0	10.0
δ, mm s^{-1}	0.42, 0.43, 0.44, 0.45	0.49, 0.62		
			0.10	1.33
	(0.41,0.42,0.43,0.44)	(0.45, 0.53)		
ΔE_Q, mm s^{-1}	0.80, 1.10, 1.20, 1.40	1.32, 1.50	0.60	3.00
	(0.70,1.00,1.10,1.30)	(0.90, 1.10)		
η	0.6, 0.6, 0.8, 0.8	0.83, 0.11		
A_x, T		-11.1, 18.8		-10
A_y,T		-28.0, 4.0		-10
A_z, T		-24.0,10.4		-10
Γ, mm s^{-1}	0.27, 0.27, 0.27, 0.27	0.5, 0.4	0.25	0.45

[a]This work, obtained from the simulations of the spectra at different applied fields perpendicular to the γ beam at 4.2 K. Isomer shifts (δ) and quadrupole splitting (ΔE_Q) shown in parenthesis correspond to 160 K. η is the asymmetry parameter, A_i are the hyperfine tensor components and Γ is the line width. g = 2.0 was taken for all the species, otherwise stated. For the Fe^{II} high spin species S = 2, D = 10 cm^{-1} and E/D = 0.33 were assumed. No oxidized $[4Fe4S]^{2+}$ cluster was considered. The slow relaxation limit was assumed at 4.2 K and fast relaxation limit was used at 160 and 80 K. Errors are estimated as follows: ± 0.01 m s^{-1} (δ),± 0.05 m s^{-1} (ΔE_Q), ± 0.1 (η) and ± 0.5 MHz (A) ± 0.5 for the intensities.

```
                 20        30        40        50        60
                  .         .         .         .         .
Ral_eu_RH       NVLWIQSGGCGGCSMSLLCADTTDFTGMLKSAGIHMLWHPSLSLESG-----------VE
Pse_ae_RH       RVLWLQSGGCGGCNMSLLCADTQDFAGLWRSAGIELLWHPSLSLESG-----------NE
Pse_ni_RH       RVLWLQSGGCGGCNMSLLCADTQDFAGLWRNAGIELLWHPSLSLESA-----------HE
Pse_ve_RH       RVLWLQSGGCGGCNMSLLCADTQDFFAMWRSAGIELLWHPSLSLESG-----------DQ
Pol_na_RH       NVLWLQSGGCGGCSMSLLCADTTDFHGHLRDAGIHLLWHPSLSLESG-----------HE
Var_pa_RH       NVLWLQSGGCGGCSMSLLCADTSDFHGQLRDAGINLLWHPSLSIESG-----------SE
Alc_hy_RH       NVLWLQSGGCGGCSMSLLCADSADFFGSLQDAGINMLWHPSLSLETG-----------AD
Bur_sp_RH       NVLWLQSGGCGGCNMSLLCADTPDFSGMLSRAGIRLLWHPSLSLETG-----------AD
Rho_fe_RH       NVLWLQSGGCGGCSMSLLCADTTDFPALLRSNGIRLLWHPALSLAGE-----------QD
Azo_do_RH       TILWLQSGGCGGCTMSLLCAEAPDLMTTLETAGIHMLWHPSLSEETG-----------AE
Bos_sp_RH       NVLWVQSGGCGGCTMSMLCAEAPDLATTLASANIRMLWHPTLSEETG-----------TE
Xan_sp_RH       TVLWLQSGGCGGCTMSLLCAEAPDLAATMASANIRFLWHPTLSEETG-----------DE
Azo_ha_RH       SVLWLQSGGCGGCTMSMLCAQSPDLPTLLEIAGIRLLWHPSLSEETG-----------AE
Oli_ca_RH       TVLWLQSGGCGGCTMSMMCAENPGLFATLENFGLDFLWHPSLSEESG-----------TE
Rho_ca_RH       KVLWLQASGCGGCTMSALCAEAPDLIDTLATAGVEFLWHPALSLATG-----------GE
Rho_sp_RH       NILWLQASGCGGCTMSLLCAEAPGLFDLLEDAGLCFLWHPSLSVESG-----------AE
Bra_di_RH       NVLWLQGASCGGCTMSILESGASGWFDELRQFGINLLWHPSVSEETG-----------EE
Des_vu          SVVYLHNAECTGCSESVLRAFEPYIDTLILDT-LSLDYHETIMAAAG-----------DA
Des_al          SVVYLHNAECTGCSESVLRAFNPYIDELLLDT-ISLDYHETIMAAAG-----------HA
Des_ba          SVVWLHNAECTGCSESILRAVRPFIDDLILDT-ISLDYHETLMAASG-----------HK
Des_re          SVVWLHNAECTGCSESVLRTVSPYIDELLLDT-ISLDYHETLMQASG-----------EA
Des_de          SVVYLHAAECTGCSEALLRTYQPFIDTLILDT-ISLDYHETIMAAAG-----------EA
All_vi          SVIWLSFQECTGCTESLTRAHAPTLEDLILDF-ISLDYHHTLQAASG-----------EA
Thi_ro          SVIWLSFQECTGCTESLTRSHAPTLEDLILDV-ISLDYHHTLQAAAG-----------DA
Thi_sp          SVIWLSFQECTGCVESLTRSYAPSLESLIFDF-ISLDYQHALQAAAG-----------HQ
Aqu_ae          PVLWIHGLECTCCSESFIRSATPLASDVVLSM-ISLEYDDTLSAAAG-----------EA
Hyd_th          PVLWIHGLECTCCSESFIRSATPLASDVVLSM-ISLEYDDTLSAAAG-----------EA
Ral_eu          PVLWLHGLECTCCSESFIRSAHPLAKDVVLSM-ISLDYDDTLMAAAG-----------HQ
Hyd_ma          PVIWLHGLECTCCSESFIRSAHPLAKDVVLSM-ISLDYDDTLMAASG-----------HA
Met_ba          KLLWIHGSECTGCSESVLNAGNPDLVQALQKLNVNLAYHETLCAQQGIWNDGELVNTSEL
Met_ma          KLIWIHGSECTGCSESLLNGGNPDVAQALTKLNVNLAYHETLCMQQGIWNDGELVNTSEL
Met_ac          KIIWLHGAECTGCSESILNGGNPDIIQAINKLNVNLAYHETLLAQQGLFVDDEPVNTSEL
                 ::::    *  *  :                 : : :. ::
```

Figure S9. Sequence alignment of selected hydrogenase small subunit sequences of H_2-sensing regulatory hydrogenases (group 2, top 17 sequences) and group 1 enzymes (membrane-bound and/or periplasmic). Only the region corresponding to residues 18-77 of the HoxB subunit of the *R. eutropha* RH is shown. The two cysteines highlighted in red are the two first residues making up the CXXC ligand motif of the [4Fe-4S] cluster proximal to the [Ni-Fe] active site. Glycine and methionine residues that are located in the vicinity of the proximal cluster at the interface of the small and large subunit of H_2-sensors are highlighted in cyan blue. These residues are conserved only in regulatory hydrogenases and may have a role in tuning the redox potential of the proximal cluster. Residues conserved within a 70% similarity score (% equivalent) are marked in yellow. Abbreviations: *Ral_eu*, *Ralstonia eutropha*; *Pse_ae*, *Pseudomonas aeruginosa*; *Pse_ni*, *Pseudomonas nitroreducens*; *Pse_ve*, *Pseudomonas veronii*, *Pol_na, Polaromonas naphthalenivorans*; *Var_pa*, *Variovorax paradoxus*; *Alc_hy*, *Alcaligenes hydrogenophilus*; *Bur_sp*, *Burkholderia sp.*; *Rho_fe*, *Rhodoferax ferrireducens*; *Azo_do*,

Azorhizobium doebereinerae; *Bos_sp*, *Bosea sp.*; *Xan_sp*, *Xanthobacter sp.*; *Azo_ha*, *Azospirillum halopraeferens*; *Oli_ca*, *Oligotropha carboxidovorans*; *Rho_ca*, *Rhodobacter capsulatus*; *Rho_sp*, *Rhodobacter sphaeroides*; *Bra_di*, *Bradyrhizobium diazoefficiens*; *Des_vu*, *Desulfovibrio vulgaris*; *Des_al*, *Desulfovibrio alaskensis*, *Des_ba*, *Desulfomicrobium baculatum*; *Des_re*, *Desulfohalobium retbaense*; *Des_de*, *Desulfovibrio desulfuricans*; *All_vi*, *Allochomatium viosum*; *Thi_ro*, *Thiocapsa roseopersicina*; *Thi_sp*, *Thioalkalivibrio sp.*; *Aqu_ae*, *Aquifex aeolicus*; *Hyd_th*, *Hydrogenobacter thermophilus*; *Hyd_ma*, *Hydrogenophilus marinus*; *Met_ba*, *Methanosarcina barkeri*; *Met_ma*, *Methanosarcina mazei*; *Met_ac*, *Methanosarcina acetivorans*.

References

(1) Gütlich, P.; Link, R.; Trautwein, A. X. *Mössbauer Spectroscopy and Transition Metal Chemistry*; Springer, 1978.

(2) Gütlich, P.; Bill, E.; Trautwein, A. X. *Mössbauer Spectroscopy and Transition Metal Chemistry:Fundamentals and Applications*; Springer-Verlag Berlin Heidelberg: Berlin, 2011.

(3) Schünemann, V.; Winkler, H. *Rep Prog Phys* **2000**, *63*, 263.

(4) Schweiger, A.; Jeschke, G. *Principles of Pulse Electron Paramagnetic Resonance*; Oxford University Press, USA, 2001.

(5) Huynh, B. H.; Patil, D. S.; Moura, I.; Teixeira, M.; Moura, J. J. G.; DerVartanian, D. V.; Czechowski, M. H.; Prickril, B. C.; Peck, H. D.; Legall, J. *J Biol Chem* **1987**, *262*, 795.

(6) Teixeira, M.; Moura, I.; Xavier, A. V.; Moura, J. J. G.; Legall, J.; DerVartanian, D. V.; Peck, H. D.; Huynh, B. H. *J Biol Chem* **1989**, *264*, 16435.

(7) Teixeira, M.; I, M.; Fauque, G.; DerVartanian, D. V.; Legall, J.; Peck, H. D.; Moura, J. J. G.; Huynh, B. H. *Eur J Biochem* **1990**, *189*, 381.

(8) Middleton, P.; Dickson, D. P. E.; Johnson, C. E.; Rush, J. D. *Eur J Biochem* **1978**, *88*, 135.
